# Discovery of a Small Molecule Inhibitor of Human Adenovirus Capable of Preventing Escape from the Endosome

**DOI:** 10.3390/ijms22041617

**Published:** 2021-02-05

**Authors:** Jimin Xu, Judith Berastegui-Cabrera, Marta Carretero-Ledesma, Haiying Chen, Yu Xue, Eric A. Wold, Jerónimo Pachón, Jia Zhou, Javier Sánchez-Céspedes

**Affiliations:** 1 Chemical Biology Program, Department of Pharmacology and Toxicology, University of Texas Medical Branch (UTMB), Galveston, TX 77555, USA; jimxu@utmb.edu (J.X.); haichen@utmb.edu (H.C.); yuxue1@utmb.edu (Y.X.); eawold@utmb.edu (E.A.W.); 2 Unit of Infectious Diseases, Microbiology and Preventive Medicine, Institute of Biomedicine of Seville (IBiS), University Hospital Virgen del Rocío, CSIC, University of Seville, E41013 Seville, Spain; jbc9393@hotmail.com (J.B.-C.); marta.carreterole@gmail.com (M.C.-L.); pachon@us.es (J.P.); 3 Department of Medicine, University of Seville, E-41009 Seville, Spain

**Keywords:** antiviral agent, adenovirus, salicylamide derivatives, entry inhibition

## Abstract

Human adenoviruses (HAdVs) display a wide range of tissue tropism and can cause an array of symptoms from mild respiratory illnesses to disseminated and life-threatening infections in immunocompromised individuals. However, no antiviral drug has been approved specifically for the treatment of HAdV infections. Herein, we report our continued efforts to optimize salicylamide derivatives and discover compound **16** (JMX0493) as a potent inhibitor of HAdV infection. Compound **16** displays submicromolar IC_50_ values, a higher selectivity index (SI > 100) and 2.5-fold virus yield reduction compared to our hit compound niclosamide. Moreover, unlike niclosamide, our mechanistic studies suggest that the antiviral activity of compound **16** against HAdV is achieved through the inhibition of viral particle escape from the endosome, which bars subsequent uncoating and the presentation of lytic protein VI.

## 1. Introduction

Human adenoviruses (HAdVs) are common pathogens with broad tissue tropism and are frequently responsible for infections displaying mild to moderate respiratory and gastrointestinal symptoms as well as conjunctivitis, notably in pediatric populations and among active duty military service members. HAdVs are characterized by a linear double-stranded DNA genome, ranging from 34–36 kb in size, surrounded by a nonenveloped, icosahedral capsid [1]. Presently, HAdVs compromise more than 100 classified serotypes distributed among seven species (HAdV A–G), which display a large variance in infection and symptom severity [2,3]. In healthy individuals with normal immune function, HAdV infections are often self-limiting and are rarely associated with severe disease. However, in immunocompromised individuals and other at-risk groups, such as solid-organ transplant (SOT) and allogenic hematopoietic stem cell transplant (allo-HSCT) recipients, HAdV infections can result in life-threatening illness and infection-related medical complications [4,5]. Pediatric allo-HSCT recipients are especially high-risk, exemplified by a frequency of infections recorded as high as 42–47% with the mortality rate up to 80% in cases with disseminated disease [4,6,7]. Additionally, following increasingly accurate molecular diagnostic technology development, HAdV infections have been found to be involved in both isolated cases and outbreaks of community-acquired pneumonia (CAP) in otherwise healthy individuals [8,9,10,11,12,13].

**Figure 1:**
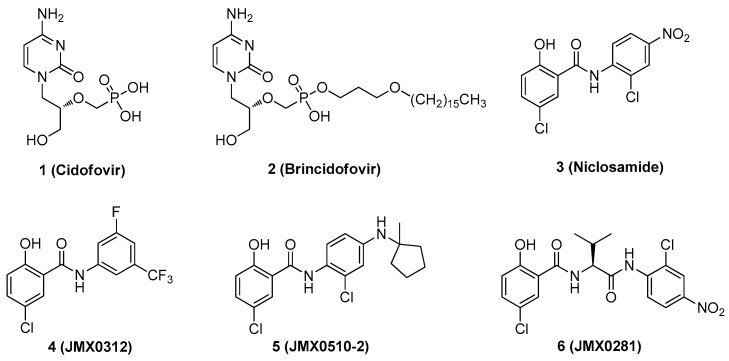
Representative human adenovirus (HAdV) inhibitors.

Despite the consequences of acquiring disseminated HAdV infection, there are no antiviral drugs specifically approved for the treatment of HAdV infections [14,15,16,17,18,19,20,21]. Currently, intravenous cidofovir (Figure 1) is used off-label to treat HAdV-associated diseases in many transplant clinics. Cidofovir, an acyclic nucleotide phosphonate cytosine analogue, is considered a broad-spectrum antiviral agent against DNA viruses and has shown efficacy against all HAdV serotypes in vitro, acting as a chain terminator during DNA replication [22]. However, cidofovir lacks sufficient oral bioavailability and is highly subject to renal elimination; thus, even its intravenous administration can result in notable nephrotoxicity and myelotoxicity, necessitating the routine coadministration of probenecid to mitigate nephrotoxicity [14]. In efforts to utilize the efficacy against HAdVs, a series of lipid ester derivatives of cidofovir were synthesized that displayed meaningfully decreased nephrotoxicity by reducing kidney exposure to the drug, and were also marked by increased oral bioavailability and cellular uptake due to the lip moiety, which is cleaved inside the cell by phospholipase to transform the prodrug into cidofovir [23,24,25]. Among these derivatives, the alkoxyalkyl ester prodrug brincidofovir (BCV, CMX001, 3-hexadecyloxy-1-propanol-cidofovir, 2) showed promising results in phase II and III clinical trials (NCT01231344 and NCT02087306), which heightened interest in its potential to be developed as the first approved treatment for HAdV infections [26,27,28,29]. Unfortunately, long-term trials of BCV treatment led to adverse effects such as diarrhea in some patients and also highlighted that alternative administration strategies should be considered [29]. Therefore, there is still an urgent need to develop highly effective antivirals against HAdV infections that are characterized by a lack of toxicity, reduced adverse effects, and increased oral bioavailability.

**Figure 2:**
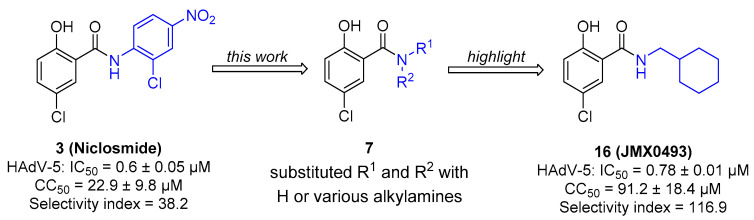
The drug design for this work, which highlights the discovery of new compound **16** (JMX0493) with both improved selectivity index and capability of inhibiting the escape from the endosome of the viral particles.

We discovered that niclosamide (3), an FDA-approved anthelmintic drug that is a part of our ongoing antiviral drug discovery and development program [30,31,32,33,34,35,36], significantly inhibited HAdV infection with an IC_50_ value of 0.6 μM, displayed moderate cytotoxicity (CC_50_ = 22.9 μM), and was characterized as having a narrow safety window, as shown by a selectivity index (SI) of 38.2 [37]. To improve potency and decrease toxicity, we focused our previous strategies for chemical modification on the aniline moiety substituents and changes to the amide linker, whereby a series of salicylamide derivatives were discovered as potent anti-HAdV inhibitors with improved safety windows [38,39]. Among these derivatives, the 3′-fluoro-5′-trifluoromethyl substituted compound **4** and the 2′-chloro-5′-(1-methylcyclopentyl)amino substituted compound **5** showed improvements in potency (IC_50_ = 0.18 μM and 0.27 μM, respectively), decreased cytotoxicity (CC_50_ = 120.0 μM and 156.8 μM, respectively) and dramatically increased the selectivity index (SI = 666.7 and 580.7, respectively), whereas compound **6** modified with a *L*-valine linker maintained the same level of potency (IC_50_ = 0.45 μM) with a decreased cytotoxicity (CC_50_ = 200.0 μM) and increased selectivity index (SI = 444.4), compared to the hit compound niclosamide. However, all of these lead compounds yielded from our previous optimization efforts shared the similarity by containing an aniline group to form the amide scaffolds like niclosamide, and whether this moiety can be altered with more conformationally flexible alkyl groups remains unclear. Herein, as depicted in Figure 2, we further explored the structure–activity relationship (SAR) studies by replacing the aniline moiety of niclosamide with various alkylamine groups to identify new antiviral agents with potential action mechanisms distinct from that of niclosamide. This work highlights the discovery of a unique compound **16** (JMX0493), which displays an improved selectivity index while acting as a potent inhibitor of HAdV infection by targeting the HAdV entry pathway that prevents viral particle disassembly and subsequent release from the endosome.

## 2. Results and Discussion

### 2.1. Chemistry

**Scheme 1:**
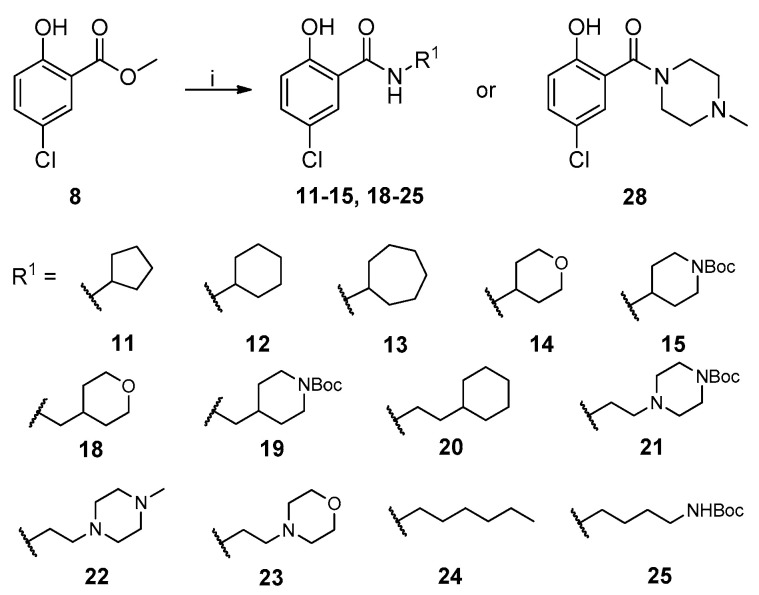
Synthetic route to achieve compounds 11–15, 18–25, and 28. Reagents and conditions: (**i**) R^1^NH_2_ or 1-methylpiperazine, CH_3_OH, r.t. to 80 °C, 48–96 h.

**Scheme 2:**
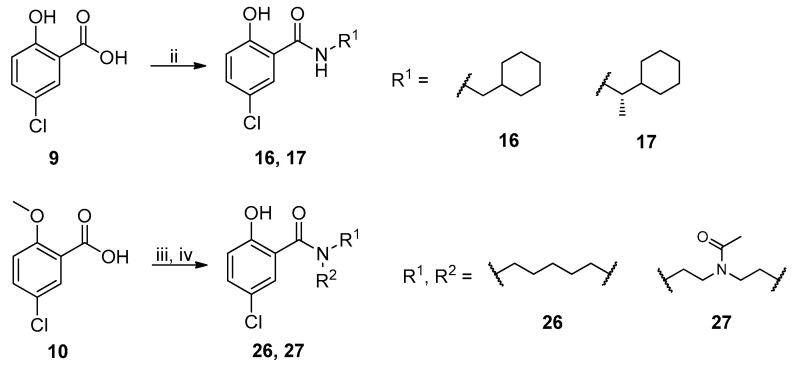
Synthetic route to achieve compounds 16, 17, 26, and 27. Reagents and conditions: (**ii**) R^1^NH_2_, EDCI, DMAP, DCM, r.t., 12 h. (**iii**) piperidine or 1-acetylpiperazine, EDCI, DMAP, DCM, r.t., 2 h; (**iv**) BBr_3_, DCM, −78 °C to 0 °C, 12 h.

The general synthesis of salicylamide derivatives is shown in Scheme 1. Direct substitution of methyl 5-chloro-2-hydroxybenzoate with a variety of amines in methanol afforded derivatives 11–15, 18–25, and 28. As shown in Scheme 2, condensation of 5-chloro-2-hydroxybenzoic acid with cyclohexylmethanamine or (*S*)-1-cyclohexylethanamine provided compounds **16** and **17**, respectively. 5-Chloro-2-methoxybenzoic acid was coupled with piperidine or 1-acetylpiperazine followed by demethylation with BBr_3_ to give compounds **26** and **27**, respectively. Efficient and accessible synthetic methodology is imperative in antiviral discovery efforts and remains a consideration in our molecular design along with robust SAR studies discussed in the following section.

### 2.2. In Vitro Evaluation of Human Adenovirus Inhibition

The newly synthesized compounds were first screened in the plaque assay, measuring the inhibition of HAdV plaque formation. As shown in Table 1, we first substituted the aniline moiety of niclosamide with simple cycloalkanamines (11–15). When we increased the ring size of the cycloalkyl groups, the corresponding compounds **11**–**13** showed a trend where ring size correlated with increased potency with inhibition values of 22.6%, 57.6%, and 99.6% at 10 μM, respectively. Compound **13** with the *N*-cycloheptyl substitution displayed micromolar potency (IC_50_ = 4.7 μM) and similar cytotoxicity (CC_50_ = 30.9 μM) to niclosamide. Interestingly, the tetrahydropyran derivative 14 was completely inactive at 10 μM, whereas the *N*-Boc-piperidine-substituted compound **15** inhibited HAdV plaque formation with a percentage of 52.2%. Inserting one carbon atom as a linker between the amide and cycloalkyl produced compounds **16**–**19**. Excitingly, the *N*-cyclohexylmethyl substituted derivative 16 exhibited submicromolar potency (IC_50_ = 0.78 μM) and, in contrast to niclosamide, displayed significantly decreased cytotoxicity (CC_50_ = 91.2 μM) and an increased selectivity index (SI = 116.9). The introduction of an (*S*)-methyl on the methylene linker (17) retained the same level of potency (IC_50_ = 0.51 μM) but significantly increased the cytotoxicity (CC_50_ = 23.0 μM) as compared with derivative 16, steering our efforts away from similar such modifications. Compound **18** with a tetrahydropyran moiety showed no anti-HAdV activity, whereas the *N*-Boc protected derivative 19 was active with an inhibition percent of 88.5% against HAdV plaque formation at 10 μM. By adding an additional carbon atom in the linker to achieve the *N*-cyclohexylethyl derivative 20, we observed a slight loss of potency (IC_50_ = 1.0 μM) but also diminished cytotoxicity (CC_50_ = 66.3 μM) and improved selectivity index (SI = 64.3) compared to niclosamide. However, building on the two-carbon linker theme, we found that neither piperazine analogue (21 and 22) nor the morpholine analogue 23 showed meaningful inhibitory activity against HAdV plaque formation up to 10 μM. The lack of inhibitory activity by 23 and 14 may be associated with limited tolerance for electronegative atoms within R^2^ position substituents and is under subsequent investigation. Surprisingly, derivative 24 with a linear hexylamine substitution maintained single digit micromolar potency with an IC_50_ value of 3.73 μM. As expected, *N*-Boc protected derivative 25 was also active against HAdV with inhibition of 74.0% at 10 μM. To probe molecular space constraints, secondary amine intramolecular ring moieties were applied but found not to be tolerated in any configuration tested, as shown by the activity data for compounds **26**–**28**. Based on our previous work and the SAR reported herein, we hypothesize that the target binding site accommodating the R^2^ moiety is preferentially engaged by simple hydrophobic moieties that are either of moderate length (e.g., one carbon linker) or shortened, bulky groups (e.g., *N*-cycloheptyl moiety), and are devoid of terminal electronegative atoms as seen in tetrahydropyran and morpholine derivatives. Larger, more complex moieties may be tolerated, but at a cost of incorporating additional functional groups that are metabolically labile or contributors to cytotoxicity. Thus, compound **16** was of keen interest due to the conservative *N*-cyclohexylmethyl substitution and its noteworthy inhibition activity, low cytotoxicity, and high selectivity index (SI > 100).

We next evaluated the anti-HAdV effect of compound **16** using a virus burst assay that can measure the ability of a pharmacological agent to suppress the production of new viral particles. As shown in Table 2, the addition of compound **16** resulted in a 208-fold overall reduction in virus yield, 2.5 times greater than the reduction observed by niclosamide addition.

### 2.3. Antiviral Mechanism of Action Studies

The HAdV replicative cycle is a multistage process characterized by numerous, low-redundancy steps that may be targeted to inhibit viral attachment, entry, replication, and/or escape. Thus, we investigated several key points in its replicative cycle to identify a potential target responsible for the antiviral activity of compound **16**.

#### 2.3.1. Impact of Compound **16** on HAdV Replication

**Figure 3:**
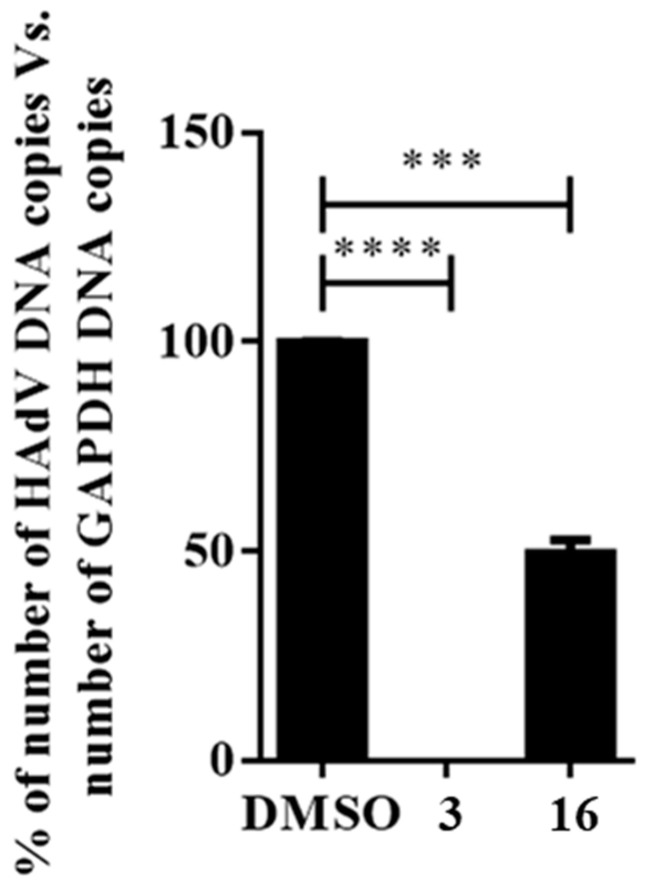
Effect of compound **16** on the HAdV DNA replication. Concentration of niclosamide (3) was 5 μM and 10-fold IC_50_ concentration obtained in the plaque assay for compound **16**. Bars represent means ± SD of triplicate samples. *** *p* ≤ 0.001, **** *p* ≤ 0.0001.

We first examined the effect that compound **16** had on the HAdV DNA replication process. As viral replication is dependent upon efficient DNA replication, we performed quantitative real-time PCR to measure HAdV DNA replication in the presence of compound **16**. After one round of infection, DNA was extracted at 24 h, avoiding the influence of newly generated viral particles derived from subsequent rounds of infection occurring at 32–36 h timepoints [19]. As shown in Figure 3, compound **16** displayed significant activity in the qPCR assay, inhibiting or delaying HAdV-5 DNA replication by 50% at a concentration of 7.8 μM (*p* ≤ 0.001) when compared to the DMSO treated control, whereas niclosamide prevented any detectable DNA replication. This result indicates that compound **16** likely targets, at least partially, the HAdV DNA replication process. The decrease in HAdV DNA copy number in the presence of compound **16** suggests that this molecule could be interfering directly with proteins involved in HAdV DNA replication or with an earlier step in its replicative cycle, through which interference would indirectly downregulate DNA replication.

#### 2.3.2. Time of Addition Assay

**Figure 4:**
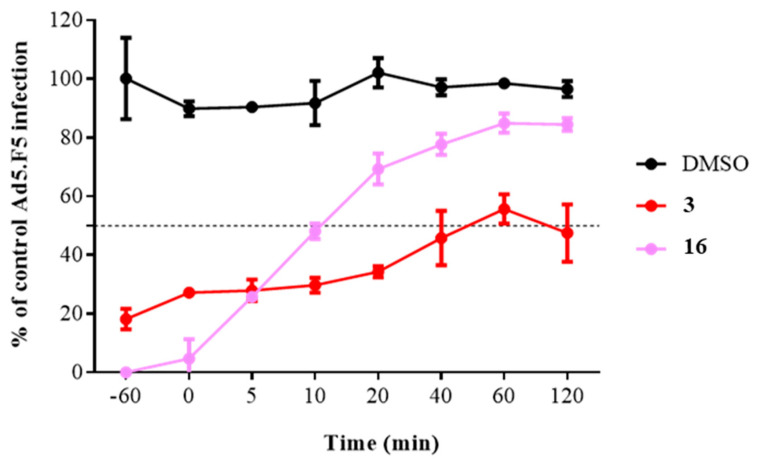
Effect of compound **16** on HAdV infection at different time points. The concentrations used were 5 μM for niclosamide (3) and 10-fold IC_50_ concentration obtained in the plaque assay for analog 16. Line charts represent means ± SD of duplicate samples.

To obtain a more complete picture and pinpoint the potential step in the HAdV replicative cycle targeted by compound **16**, we measured the time dependence of compound addition on its ability to inhibit HAdV. HAdV has been shown to be enter the cell within 5 min of viral attachment, to escape the endosome after 15 min, and to attach to the nuclear pore complex after 35−45 min postinfection (p.i.). Our results showed that the antiviral activity of compound **16** exhibited a time-dependent decrease in activity Figure 4, especially apparent at early time points where we observed a decrease in its inhibitory activity from 95.3% inhibition at 0 min p.i. to 30.7% inhibition at 20 min p.i. Collectively, these results indicate that compound **16** inhibits an early step in the virus cycle, after cell attachment and before entry into the cell nucleus.

**Figure 5:**
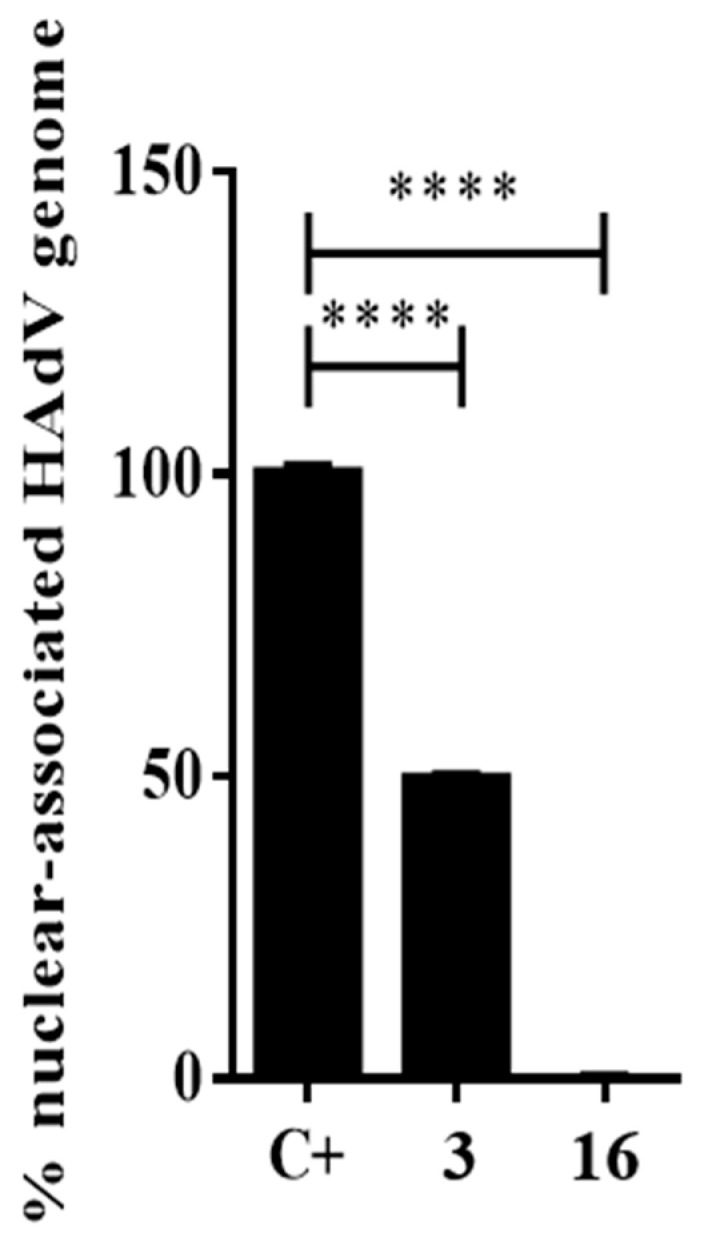
Percentage of nuclear-associated HAdV genome of compound **16**. Concentration of niclosamide (3) was 5 μM and 10-fold IC_50_ concentration obtained in the plaque assay for compound **16**. Bars represent means ± SD of triplicate samples. **** *p* ≤ 0.0001.

To confirm if the antiviral activity of compound **16** was dependent on a step between HAdV entry and the arrival to the nucleus, we performed an assay to quantify the HAdV genome accessibility to the nucleus. Once HAdV escapes from the endosome, the partially uncoated capsid is transported to the cell nucleus, where further disassembly occurs and the HAdV genome is released into the cell nucleus for early gene transcription and subsequent replication. Thus, any inhibitory effect on that process will be reflected by the number of HAdV genomes that reach the host nucleus after a synchronized infection. Briefly, 45 min after infection, cells were recovered to separate the cytoplasmic and nuclear fractions. Then, HAdV DNA was isolated from each fraction and quantify by the real-time PCR. As shown in Figure 5, compound **16** inhibited more than 99% of the HAdV DNA accessibility to the nucleus and showed a higher inhibitory effect compared to the DMSO control experiment and niclosamide at 45 min postinfection (*p* ≤ 0.0001). At this point, our mechanistic approaches confirmed that the antiviral activity of compound **16** was targeting a step before HAdV transcription of early genes and DNA replication. Going backwards in the HAdV replicative cycle, the next process is to identify the precise step targeted by compound **16** through evaluating the HAdV escape from the endosome.

#### 2.3.3. Impacts of Compound **16** on HAdV Escape from the Endosome

**Figure 6:**
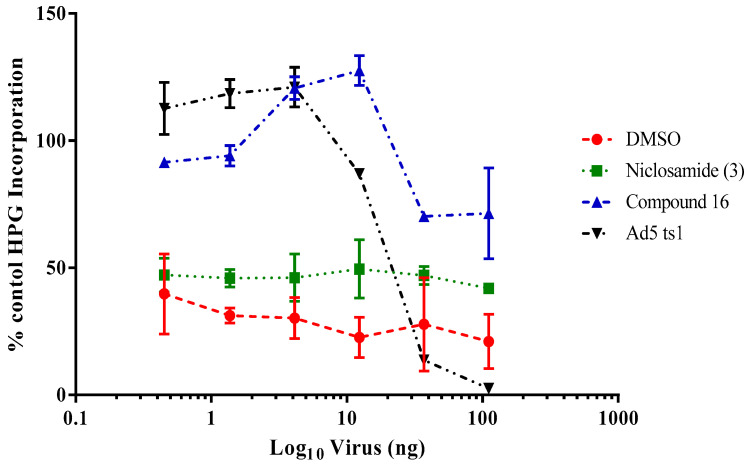
Activity of compound **16** on HAdV-mediated endosomolysis in a α-sarcin assay. The concentrations used were 5 μM for niclosamide (3) and 10-fold IC_50_ concentration obtained in the plaque assay for derivative 16. Results represent means ± SD of triplicate assays.

Upon attachment to its cellular receptors, HAdV particles are internalized by endocytosis into the cells and viral particles undergo partial disassembly inside the early endosomes, resulting in the release of protein VI from the interior of the capsid, which plays a key role in HAdV escape from the endosome [40]. To evaluate the potential influence of compound **16** in preventing the HAdV escape from the endosome, we used the α-sarcin co-delivery assay as a measurement of the ability of this compound to interfere with virus-mediated endosome lysis. Since the α-sarcin alone is unable to penetrate the cell, in this assay, the successful virus-mediated lysis of the endosome would result in ribotoxin-mediated inhibition of cellular protein synthesis. Increasing concentrations of HAdV in the presence or absence of compound **16** were mixed with α-sarcin and incubated with A549 cells. After metabolic labeling with methionine L-homopropargylglycine (HPG), cell samples were assayed for HPG incorporation into cellular proteins. As shown in Figure 6, cells treated with either the vehicle (DMSO) or niclosamide showed rates of HPG incorporation under 50% compared to a control without α-sarcin, independent of the virus concentration used. Interestingly, compound **16** showed a similar behavior as the entry-defective *ts1* mutant HAdV that contains a mutation in the protease gene and fails to penetrate cell endosomes [41]. Compound **16** prevented HAdV-mediated endosome lysis at concentrations of the virus below 12 ng, similar to the effects observed by the *ts1* mutant. This result, together with our time of addition assay, showing a decrease of the inhibitory effect after 15–20 min p.i, suggests that the mechanism for inhibition of this compound is likely related to the blockage of HAdV escape from the endosome.

**Figure 7:**
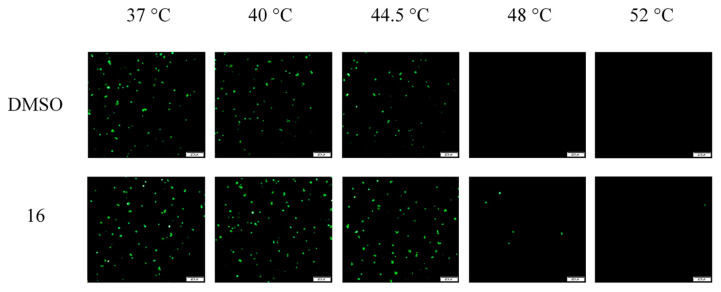
Compound **16** stabilizes HAdV capsid. The concentration used was 50 μM. Data are representative of three experiments.

The studies described above delineate the step of infection that is targeted by compound **16**; however, the precise molecular mechanism involved in its antiviral activity remained to be established. One alternative mode of action was that compound **16** could block HAdV-mediated lysis of the endosome by stabilizing the virus capsid, thereby preventing uncoating. Indeed, human defensins and neutralizing antibodies have been previously shown to act through this mechanism [42]. To determine whether compound **16** impacts HAdV uncoating, we used a thermostability assay that mimics virus disassembly in the endosome, which was previously described by Wiethoff et al. [40], with a few modifications. In this assay, temperatures above 48 °C promote selective removal of the virus vertex region. HAdV-5 was incubated with or without 50 µM concentrations of compound **16** at temperatures from 37 °C to 52 °C and then added to cells to evaluate the viability of the viruses. Viruses incubated with either compound **16** or DMSO at 37 °C, 40 °C, and 44.5 °C were largely intact, showing similar rates of infection measured by the number of cells expressing GFP Figure 7. Upon heating HAdV-5 to 48 °C or above, in those nontreated with compound **16**, there was no GFP expression 24 h postinfection. However, in wells with the addition of 16, significant levels of GFP expression were observed for those viruses heated to 48 °C, and some residual expression remained at 52 °C. These findings suggest that compound **16** may prevent HAdV endosomal escape by stabilizing the viral capsid and subsequently inhibiting endosomal membrane lysis [43].

### 2.4. Synergistic Activity of the Selected Compounds

In previous reports [38,39], we found three compounds—**4** (JMX0312), **5** (JMX0510-2), and **6** (JMX0281)—with high activity against HAdV, targeting different steps in the HAdV replicative cycle. We hypothesize that the double and triple combination of these compounds with compound **16** (JMX0493) should significantly increase their antiviral activity. To evaluate our hypothesis, we conducted a combination study based on the Chou–Talalay method for drug combination using the CalcuSyn software [44]. The constant ratio for each combination was selected based on the IC_50_ values for each drug. The data for all the combinations showed good conformity with the mass action law principle (*r* = 0.87–0.95) Table 3. Two out of three double combinations showed a synergistic effect, and concretely, the combination of compounds **4** and **16** (ratio 1:4.3) showed strong synergism (ED_50_ = 0.26) and the combination of compounds **6** and **16** (ratio 1:1.73) was also classified as synergistic (ED_50_ = 0.40), while the combination of compounds **5** and **16** (ratio 1:2.9) showed only an additive effect (ED_50_ = 1.09). For the triple combinations, one of them (the combination of compounds **4**, **5**, and **16**; ratio 1:1.5:4.3) exhibited a synergistic effect with an ED_50_ value of 0.33, while the other triple combination (compounds **5**, **6**, and **16**; ratio 1:1.7:2.9) showed a clear antagonistic effect (ED_50_ = 2.01), probably due to compound interactions. It is worth noting that in our previous report, the combinatory index also increased when compound **5** was added to the mix, reaching additive or antagonistic values.

### 2.5. Impact of Compound **16** on HCMV Replication

**Figure 8:**
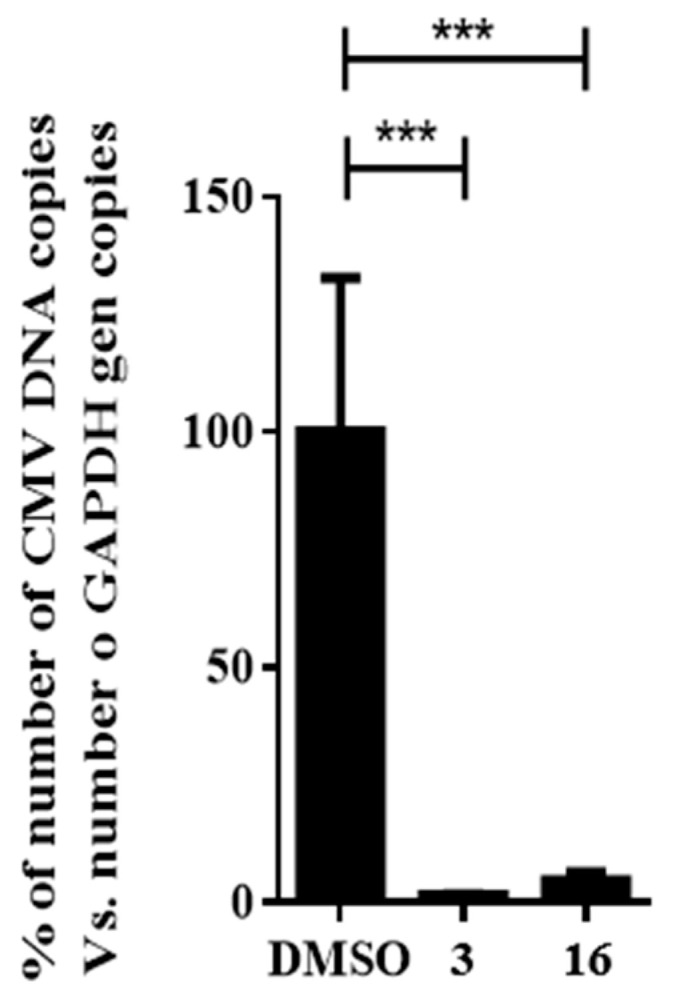
Effect of compound **16** on HCMV replication. Concentration of niclosamide (3) was 5 μM and compound **16** was tested at 10-fold IC_50_ concentration obtained in the HAdV plaque assay. Bars represent means ± SD of triplicate samples. *** *p* ≤ 0.001.

The broad antiviral activity of compound **16** was tested using human cytomegalovirus (HCMV) in a DNA replication assay. To avoid the influence of newly generated viral particles from subsequent rounds of infection, HCMV DNA was extracted from HFF cells at 72 h postinfection and quantified in a single round of infection as a measurement of the DNA replication efficiency. The addition of compound **16** displayed significant reductions (*p* ≤ 0.001) in the quantification of total HCMV DNA, showing a 95% decrease compared to the DMSO control, as depicted in Figure 8.

## 3. Conclusions

In summary, salicylamide derivatives were designed, synthesized, and biologically evaluated for their antiviral activity against HAdV. Among these new molecules, compound **16** (JMX0493) maintained submicromolar potency against HAdV while displaying a markedly higher selectivity index (SI > 100) compared to the hit compound niclosamide. In addition, compound **16** was able to reduce virus yield to a 2.5-fold greater extent than niclosamide, indicative of a safer and more effective profile in virus yield reduction. Moreover, compound **16** demonstrated potential as a broad antiviral agent by showing a significant inhibition of HCMV DNA replication. As for its mechanism of action, unlike niclosamide, compound **16** showed a specific inhibition of HAdV infection via blocking the escape of viral particles from the endosome by stabilizing the viral capsid and preventing HAdV uncoating, thereby blocking subsequent viral migration to the nuclear membrane.

## 4. Materials and Methods 

### 4.1. Chemistry

All commercially available starting materials and solvents were reagent grade and used without further purification. Reactions were performed under a nitrogen atmosphere in dry glassware with magnetic stirring. Preparative column chromatography was performed using silica gel 60, particle size 0.063–0.200 mm (70–230 mesh, flash). Analytical TLC was carried out employing silica gel 60 F254 plates (Merck, Darmstadt). Visualization of the developed chromatograms was performed with detection by UV (254 nm). NMR spectra were recorded on a Brucker-300 (^1^H, 600 and 300 MHz; ^13^C, 150 and 75 MHz) spectrometer. ^1^H and ^13^C NMR spectra were recorded with TMS as an internal reference. ^1^H and ^13^C NMR spectra of representative compounds are shown in Supplemental Figures (page S2–S19 in Supporting Information). Chemical shifts were expressed in ppm, and *J* values were given in Hz. High-resolution mass spectra (HRMS) were obtained using a Thermo Fisher LTQ Orbitrap Elite mass spectrometer. Parameters include the following: nano-ESI spray voltage was 1.8 kV; capillary temperature was 275 °C and the resolution was 60,000; ionization was achieved by positive mode. Melting points were measured on a Thermo Scientific Electrothermal Digital Melting Point Apparatus and were uncorrected. Purities of final compounds were established using analytical HPLC, which was carried out on a Shimadzu HPLC system (model: CBM-20A LC-20AD SPD-20A UV/VIS). HPLC analysis conditions: Waters μBondapak C18 (300 × 3.9 mm); flow rate 0.5 mL/min; UV detection at 270 and 254 nm; linear gradient from 10% acetonitrile in water to 100% acetonitrile in water in 20 min followed by 30 min of the last-named solvent (0.1% TFA was added into both acetonitrile and water). All biologically evaluated compounds are >95% pure.

General procedure A. Methyl 5-chloro-2-hydroxybenzoate (1.0 eq) was dissolved in methanol (10 mL/0.5 mmol) followed by addition of different amine (3.0 eq). The resulting mixture was stirred at r.t. ~80 °C for 48~96 h, and then concentrated. The residue was purified by preparative TLC to afford the final amide products.

5-Chloro-*N*-cyclopentyl-2-hydroxybenzamide (11). Compound **11** (116 mg, 90%) was prepared as a beige solid according to general procedure A (60 °C, 48 h), starting from methyl 5-chloro-2-hydroxybenzoate and cyclopentylamine. HPLC purity 99.9% (*t*_R_ = 18.07 min). ^1^H NMR (300 MHz, CDCl_3_) δ 12.34 (s, 1H), 7.37–7.26 (m, 2H), 6.90 (d, *J* = 8.7 Hz, 1H), 6.39 (d, *J* = 4.2 Hz, 1H), 4.45–4.27 (m, 1H), 2.21–1.98 (m, 2H), 1.83–1.45 (m, 6H). ^13^C NMR (75 MHz, CDCl_3_) δ 168.6, 160.0, 133.9, 125.2, 123.3, 120.1, 115.5, 51.8, 33.1 (2C), 23.9 (2C). HRMS (ESI) calculated for C_12_H_15_ClNO_2_, 240.0791 (M + H)^+^; found, 240.0785.

5-Chloro-*N*-cyclohexyl-2-hydroxybenzamide (12). Compound **12** (71 mg, 51%) was prepared as a beige solid according to general procedure A (60 °C, 48 h), starting from methyl 5-chloro-2-hydroxybenzoate and cyclohexylamine. HPLC purity 99.9% (*t*_R_ = 18.85 min). ^1^H NMR (300 MHz, CDCl_3_) δ 12.36 (s, 1H), 7.35–7.27 (m, 2H), 6.90 (d, *J* = 8.4 Hz, 1H), 6.21 (d, *J* = 5.4 Hz, 1H), 4.00–3.86 (m, 1H), 2.06–1.94 (m, 2H), 1.83–1.60 (m, 3H), 1.48–1.13 (m, 5H). ^13^C NMR (75 MHz, CDCl_3_) δ 168.1, 160.2, 134.0, 125.1, 123.3, 120.2, 115.6, 49.0, 33.0 (2C), 25.5, 25.0 (2C). HRMS (ESI) calculated for C_13_H_17_ClNO_2_, 254.0948 (M + H)^+^; found, 254.0941.

5-Chloro-*N*-cycloheptyl-2-hydroxybenzamide (13). Compound **13** (75 mg, 52%) was prepared as an off-white solid according to general procedure A (60 °C, 48 h), starting from methyl 5-chloro-2-hydroxybenzoate and cycloheptylamine. HPLC purity 99.5% (*t*_R_ = 19.58 min). ^1^H NMR (300 MHz, CDCl_3_) δ 12.36 (s, 1H), 7.34–7.27 (m, 2H), 6.90 (d, *J* = 9.0 Hz, 1H), 6.27 (d, *J* = 5.7 Hz, 1H), 4.20–4.03 (m, 1H), 2.08–1.93 (m, 2H), 1.74–1.47 (m, 10H). ^13^C NMR (75 MHz, CDCl_3_) δ 167.8, 160.2, 133.9, 125.1, 123.3, 120.2, 115.6, 51.2, 35.1 (2C), 28.0 (2C), 24.2 (2C). HRMS (ESI) calculated for C_14_H_19_ClNO_2_, 268.1104 (M + H)^+^; found, 268.1097.

5-Chloro-2-hydroxy-*N*-(tetrahydro-2*H*-pyran-4-yl)benzamide (14). Compound **14** (30 mg, 22%) was prepared as a light-yellow solid according to general procedure A (80 °C, 96 h), starting from methyl 5-chloro-2-hydroxybenzoate and 4-aminotetrahydropyran. HPLC purity 99.9% (*t*_R_ = 15.48 min). ^1^H NMR (300 MHz, CDCl_3_) δ 12.18 (s, 1H), 7.38–7.29 (m, 2H), 6.98–6.88 (m, 1H), 6.21 (d, *J* = 6.6 Hz, 1H), 4.26–4.12 (m, 1H), 4.07–3.96 (m, 2H), 3.59–3.46 (m, 2H), 2.05–1.91 (m, 2H), 1.69–1.52 (m, 2H). ^13^C NMR (75 MHz, CDCl_3_) δ 168.4, 160.3, 134.3, 125.1, 123.4, 120.3, 115.3, 66.8 (2C), 46.5, 33.1 (2C). HRMS (ESI) calculated for C_12_H_15_ClNO_3_, 256.0740 (M + H)^+^; found, 256.0734.

*tert*-Butyl 4-(5-chloro-2-hydroxybenzamido)piperidine-1-carboxylate (15). Compound **15** (24 mg, 13%) was prepared as an off-white solid according to general procedure A (80 °C, 96 h), starting from methyl 5-chloro-2-hydroxybenzoate and 4-aminopiperidine-1-carboxylic acid *tert*-butyl ester. HPLC purity 98.8% (*t*_R_ = 18.51 min). ^1^H NMR (300 MHz, CDCl_3_) δ 12.21 (s, 1H), 7.43–7.23 (m, 2H), 6.96–6.88 (m, 1H), 6.44 (d, *J* = 6.6 Hz, 1H), 4.20–4.02 (m, 3H), 2.97–2.80 (m, 2H), 2.05–1.94 (m, 2H), 1.52–1.40 (m, 11H). ^13^C NMR (75 MHz, CDCl_3_) δ 168.5, 160.3, 154.9, 134.2, 125.3, 123.4, 120.3, 115.4, 80.1, 47.5, 42.9 (2C), 32.0 (2C), 28.6 (3C). HRMS (ESI) calculated for C_17_H_24_ClN_2_O_4_, 355.1425 (M + H)^+^; found, 355.1417.

5-Chloro-*N*-(cyclohexylmethyl)-2-hydroxybenzamide (16). To a solution of cyclohexanemethylamine (200 mg, 1.77 mmol), 5-chlorosalicylic acid (244 mg, 1.41 mmol) and DMAP (18 mg, 0.14 mmol) in 20 mL of DCM were added EDCI (509 mg, 2.66 mmol) at 0 °C. The resulting mixture was stirred at r.t. for 24 h and then concentrated. The residue was purified by column chromatography (Hex/EtOAc = 10/1) to afford compound **16** (240 mg, 63%) as a light-yellow solid. HPLC purity 99.9% (*t*_R_ = 19.68 min). ^1^H NMR (300 MHz, CDCl_3_) δ 12.22 (brs, 1H), 7.36–7.28 (m, 2H), 6.92 (d, *J* = 8.4 Hz, 1H), 6.43 (s, 1H), 3.28 (t, *J* = 6.3 Hz, 2H), 1.82–1.50 (m, 6H), 1.34–1.10 (m, 3H), 1.06–0.90 (m, 2H). ^13^C NMR (75 MHz, CDCl_3_) δ 169.0, 160.2, 134.0, 125.0, 123.4, 120.2, 115.5, 46.1, 38.0, 31.0 (2C), 26.4, 25.9 (2C). HRMS (ESI) calculated for C_14_H_19_ClNO_2_, 268.1104 (M + H)^+^; found, 268.1101.

(*S*)-5-Chloro-*N*-(1-cyclohexylethyl)-2-hydroxybenzamide (17). Compound **17** was prepared using a procedure similar to that used to prepare compound **16** starting from 5-chlorosalicylic acid and (*S*)-1-cyclohexylethanamine. The title compound was obtained (138 mg, 39%) as an off-white solid. HPLC purity 99.6% (*t*_R_ = 20.25 min). ^1^H NMR (300 MHz, CDCl_3_) δ 12.36 (s, 1H), 7.33 (dd, *J* = 8.7, 2.4 Hz, 1H), 7.29 (d, *J* = 2.4 Hz, 1H), 6.93 (d, *J* = 8.7 Hz, 1H), 6.03 (d, *J* = 7.5 Hz, 1H), 4.12–3.98 (m, 1H), 1.85–1.61 (m, 5H), 1.50–1.38 (m, 1H), 1.32–0.95 (m, 8H). ^13^C NMR (75 MHz, CDCl_3_) δ 168.3, 160.4, 134.0, 124.8, 123.3, 120.3, 115.6, 50.1, 43.2, 29.4, 29.2, 26.4, 26.2, 26.2, 18.0. HRMS (ESI) calculated for C_15_H_21_ClNO_2_, 282.1261 (M + H)^+^; found, 282.1257.

5-Chloro-2-hydroxy-*N*-((tetrahydro-2*H*-pyran-4-yl)methyl)benzamide (18). Compound **18** (117 mg, 80%) was prepared as a light-yellow solid according to general procedure A (r.t., 48 h), starting from methyl 5-chloro-2-hydroxybenzoate and 4-(aminomethyl)tetrahydropyran. HPLC purity 99.8% (*t*_R_ = 16.12 min). ^1^H NMR (300 MHz, CDCl_3_) δ 12.23 (s, 1H), 7.45 (d, *J* = 2.4 Hz, 1H), 7.31 (dd, *J* = 9.0, 2.4 Hz, 1H), 7.06 (s, 1H), 6.91 (d, *J* = 9.0 Hz, 1H), 4.05–3.94 (m, 2H), 3.49–3.27 (m, 4H), 1.99–1.81 (m, 1H), 1.73–1.61 (m, 2H), 1.47–1.29 (m, 2H). ^13^C NMR (75 MHz, CDCl_3_) δ 169.1, 159.8, 134.0, 125.5, 123.5, 120.0, 115.6, 67.6 (2C), 45.4, 35.2, 30.6 (2C). HRMS (ESI) calculated for C_13_H_17_ClNO_3_, 270.0897 (M + H)^+^; found, 270.0890.

*tert*-Butyl 4-((5-chloro-2-hydroxybenzamido)methyl)piperidine-1-carboxylate (19). Compound **19** (74 mg, 37%) was prepared as an off-white solid according to general procedure A (r.t., 48 h), starting from methyl 5-chloro-2-hydroxybenzoate and 4-(aminomethyl)-1-*N*-Boc-piperidine. HPLC purity 99.8% (*t*_R_ = 18.83 min). ^1^H NMR (300 MHz, CDCl_3_) δ 12.23 (s, 1H), 7.44 (d, *J* = 2.1 Hz, 1H), 7.30 (dd, *J* = 8.7, 2.1 Hz, 1H), 7.08–6.86 (m, 2H), 4.20–4.02 (m, 2H), 3.32 (s, 2H), 2.79–2.59 (m, 2H), 1.87–1.67 (m, 3H), 1.45 (s, 9H), 1.25–1.09 (m, 2H). ^13^C NMR (75 MHz, CDCl_3_) δ 169.2, 160.1, 155.0, 134.1, 125.5, 123.5, 120.1, 115.6, 79.8, 45.2, 43.7 (2C), 36.4, 30.0 (2C), 28.6 (3C). HRMS (ESI) calculated for C_18_H_26_ClN_2_O_4_, 369.1581 (M + H)^+^; found, 369.1572.

5-Chloro-*N*-(2-cyclohexylethyl)-2-hydroxybenzamide (20). Compound **20** (69 mg, 46%) was prepared as an off-white solid according to general procedure A (r.t., 48 h), starting from methyl 5-chloro-2-hydroxybenzoate and 2-cyclohexylethylamine. HPLC purity 99.9% (*t*_R_ = 20.07 min). ^1^H NMR (300 MHz, CDCl_3_) δ 12.33 (s, 1H), 7.38–7.27 (m, 2H), 6.91 (d, *J* = 8.7 Hz, 1H), 6.37 (s, 1H), 3.51–3.39 (m, 2H), 1.80–1.60 (m, 5H), 1.56–1.44 (m, 2H), 1.39–1.14 (m, 4H), 1.02–0.84 (m, 2H). ^13^C NMR (75 MHz, CDCl_3_) δ 169.0, 160.1, 134.0, 125.1, 123.4, 120.2, 115.5, 37.9, 36.9, 35.5, 33.2 (2C), 26.5, 26.2 (2C). HRMS (ESI) calculated for C_15_H_21_ClNO_2_, 282.1261 (M + H)^+^; found, 282.1253.

*tert*-Butyl 4-(2-(5-chloro-2-hydroxybenzamido)ethyl)piperazine-1-carboxylate (21). Compound **21** (88 mg, 43%) was prepared as an off-white solid according to general procedure A (r.t., 48 h), starting from methyl 5-chloro-2-hydroxybenzoate and 4-*N*-(2-aminoethyl)-1-*N*-Boc-piperazine. HPLC purity 98.4% (*t*_R_ = 15.08 min). ^1^H NMR (300 MHz, CDCl_3_) δ 7.35–7.25 (m, 2H), 7.15 (s, 1H), 6.89 (d, *J* = 8.7 Hz, 1H), 3.56–3.39 (m, 6H), 2.59 (t, *J* = 6.0 Hz, 2H), 2.49–2.38 (m, 4H), 1.44 (s, 9H). ^13^C NMR (75 MHz, CDCl_3_) δ 168.9, 160.1, 154.8, 134.0, 125.4, 123.3, 120.1, 115.5, 80.0, 56.3, 52.8 (2C), 43.8 (2C), 36.1, 28.5 (3C). HRMS (ESI) calculated for C_18_H_27_ClN_3_O_4_, 384.1690 (M + H)^+^; found, 384.1680.

5-Chloro-2-hydroxy-*N*-(2-(4-methylpiperazin-1-yl)ethyl)benzamide (22). Compound **22** (62 mg, 37%) was prepared as a pale solid according to general procedure A (r.t., 48 h), starting from methyl 5-chloro-2-hydroxybenzoate and 2-(4-methylpiperazin-1-yl)ethylamine. HPLC purity 95.0% (*t*_R_ = 11.25 min). ^1^H NMR (300 MHz, CDCl_3_) δ 9.45 (br s, 1H, 7.34 (d, *J* = 2.4 Hz, 1H), 7.29 (dd, *J* = 9.0, 2.7 Hz, 1H), 7.20 (s, 1H), 6.88 (d, *J* = 9.0 Hz, 1H), 3.53–3.44 (m, 2H), 2.70–2.33 (m, 10H), 2.29 (s, 3H). ^13^C NMR (75 MHz, CDCl_3_) δ 168.7, 160.0, 133.9, 125.6, 123.3, 120.0, 115.7, 56.0, 55.2 (2C), 52.8 (2C), 46.1, 36.0. HRMS (ESI) calculated for C_14_H_21_ClN_3_O_2_, 298.1322 (M + H)^+^; found, 298.1314.

5-Chloro-2-hydroxy-*N*-(2-morpholinoethyl)benzamide (23). Compound **23** (60 mg, 39%) was prepared as a grey solid according to general procedure A (r.t., 96 h), starting from methyl 5-chloro-2-hydroxybenzoate and 4-(2-aminoethyl)morpholine. HPLC purity 99.6% (*t*_R_ = 11.66 min). ^1^H NMR (300 MHz, CDCl_3_) δ 7.35–7.28 (m, 2H), 7.03 (s, 1H), 6.94–6.88 (m, 1H), 3.74 (t, *J* = 4.5 Hz, 4H), 3.51 (t, *J* = 5.7 Hz, 2H), 2.61 (t, *J* = 6.0 Hz, 2H), 2.51 (t, *J* = 4.5 Hz, 4H). ^13^C NMR (75 MHz, CDCl_3_) δ 168.9, 160.2, 134.1, 125.3, 123.3, 120.2, 115.4, 67.1 (2C), 56.5, 53.4 (2C), 35.8. HRMS (ESI) calculated for C_13_H_18_ClN_2_O_3_, 285.1006 (M + H)^+^; found, 285.0999.

5-Chloro-*N*-hexyl-2-hydroxybenzamide (24). Compound **24** (107 mg, 78%) was prepared as a white solid according to general procedure A (r.t., 48 h), starting from methyl 5-chloro-2-hydroxybenzoate and hexylamine. HPLC purity 98.0% (*t*_R_ = 19.72 min). ^1^H NMR (300 MHz, CDCl_3_) δ 12.33 (s, 1H), 7.35 (d, *J* = 2.4 Hz, 1H), 7.30 (dd, *J* = 8.7, 2.4 Hz, 1H), 6.90 (d, *J* = 8.7 Hz, 1H), 6.52 (s, 1H), 3.46–3.36 (s, 2H), 1.67–1.53 (m, 2H), 1.42–1.20 (m, 6H), 0.92–0.82 (m, 3H). ^13^C NMR (75 MHz, CDCl_3_) δ 169.0, 160.0, 134.0, 125.2, 123.4, 120.1, 115.5, 40.1, 31.5, 29.4, 26.7, 22.6, 14.1. HRMS (ESI) calculated for C_13_H_19_ClNO_2_, 256.1104 (M + H)^+^; found, 256.1098.

*tert*-Butyl (4-(5-chloro-2-hydroxybenzamido)butyl)carbamate (25). Compound **25** (56 mg, 30%) was prepared as a white solid according to general procedure A (r.t., 48 h), starting from methyl 5-chloro-2-hydroxybenzoate and *N*-Boc-1,4-butanediamine. HPLC purity 98.5% (*t*_R_ = 17.75 min). ^1^H NMR (300 MHz, CDCl_3_) δ 12.53 (s, 1H), 7.63 (s, 1H), 7.53 (s, 1H), 7.29 (dd, *J* = 8.7, 2.4 Hz, 1H), 6.90 (d, *J* = 8.7 Hz, 1H), 4.77 (s, 1H), 3.48 (q, *J* = 6.0 Hz, 2H), 3.16 (q, *J* = 6.3 Hz, 2H), 1.72–1.53 (m, 4H), 1.45 (s, 9H). ^13^C NMR (75 MHz, CDCl_3_) δ 169.3, 160.2, 156.9, 133.9, 125.9, 123.4, 119.9, 115.6, 79.9, 39.9, 39.8, 28.6, 28.6 (3C), 25.2. HRMS (ESI) calculated for C_16_H_24_ClN_2_O_4_, 343.1425 (M + H)^+^; found, 343.1417.

(5-Chloro-2-hydroxyphenyl)(piperidin-1-yl)methanone (26). To a solution of 5-chloro-2-methoxybenzoic acid (210 mg, 1.13 mmol), piperidine (80 mg, 0.94 mmol) and DMAP (28 mg, 0.23 mmol) in DCM (20 mL) was added EDCI (433 mg, 2.26 mmol) at 0 °C. The resulting mixture was stirred at r.t. for 12 h and concentrated. The residue was purified by column chromatography to afford the amide intermediate (5-chloro-2-methoxyphenyl)(piperidin-1-yl)methanone (230 mg, 96%) as colorless oil. ^1^H NMR (300 MHz, CDCl_3_) δ 7.25–7.09 (m, 2H), 6.77 (d, *J* = 8.7 Hz, 1H), 3.74 (s, 3H), 3.69–3.52 (m, 2H), 3.19–3.03 (m, 2H), 1.69–1.31 (m, 6H). ^13^C NMR (75 MHz, CDCl_3_) δ 165.9, 153.8, 129.7, 127.8, 127.5, 125.7, 112.2, 55.8, 47.9, 42.5, 26.3, 25.5, 24.5.

The amide intermediate (5-chloro-2-methoxyphenyl)(piperidin-1-yl)methanone (230 mg, 0.91 mmol) was dissolved in DCM (50 mL), and then BBr_3_ (4.53 mL, 4.53 mmol, 1 M in DCM) was added at 0 °C. The mixture was stirred at r.t. for 2 h. The mixture was diluted with DCM, washed with H_2_O and brine, dried (Na_2_SO_4_) and concentrated. The residue was purified by preparative TLC to afford compound **26** (203 mg, 93%) as a white solid. HPLC purity 95.1% (*t*_R_ = 15.13 min). ^1^H NMR (300 MHz, CDCl_3_) δ 9.57 (s, 1H), 7.26 (dd, *J* = 8.7, 2.7 Hz, 1H), 7.19 (d, *J* = 2.7 Hz, 1H), 6.93 (d, *J* = 8.7 Hz, 1H), 3.67–3.60 (m, 4H), 1.77–1.61 (m, 6H). ^13^C NMR (75 MHz, CDCl_3_) δ 169.4, 157.5, 132.3, 127.8, 123.4, 119.6, 118.9, 47.0 (2C), 26.2 (2C), 24.6. HRMS (ESI) calculated for C_12_H_15_ClNO_2_, 240.0791 (M + H)^+^; found, 240.0786.

1-(4-(5-Chloro-2-hydroxybenzoyl)piperazin-1-yl)ethanone (27). Compound **27** was prepared using a procedure similar to that used to prepare compound **26** starting from 5-chlorosalicylic acid and 1-acetylpiperazine. The title compound was obtained (139 mg, 91% in two steps) as a yellow solid. HPLC purity 97.2% (*t*_R_ = 12.32 min). ^1^H NMR (300 MHz, CDCl_3_) δ 9.51 (s, 1H), 7.23–7.14 (m, 2H), 6.86 (d, *J* = 8.7 Hz, 1H), 3.72–3.48 (m, 8H), 2.11 (s, 3H). ^13^C NMR (75 MHz, CDCl_3_) δ 169.6, 168.9, 155.0, 132.0, 127.9, 124.2, 120.8, 118.8, 46.1, 45.3, 44.8, 41.5, 21.4. HRMS (ESI) calculated for C_13_H_16_ClN_2_O_3_, 283.0849 (M + H)^+^; found, 283.0843.

(5-Chloro-2-hydroxyphenyl)(4-methylpiperazin-1-yl)methanone (28). Compound **28** (39 mg, 28%) was prepared as a pale yellow solid solid according to general procedure A (60 °C, 96 h), starting from methyl 5-chloro-2-hydroxybenzoate and 1-methylpiperazine. HPLC purity 98.3% (*t*_R_ = 10.32 min). ^1^H NMR (300 MHz, CDCl_3_) δ 7.23 (dd, *J* = 8.7, 2.7 Hz, 1H), 7.17 (d, *J* = 2.7 Hz, 1H), 6.87 (d, *J* = 8.7 Hz, 1H), 3.69 (t, *J* = 5.1 Hz, 4H), 2.45 (t, *J* = 5.1 Hz, 4H), 2.32 (s, 3H). ^13^C NMR (75 MHz, CDCl_3_) δ 169.2, 156.7, 132.2, 127.8 (2C), 123.7, 119.4, 55.0 (2C), 46.0, 45.5 (2C). HRMS (ESI) calculated for C_12_H_16_ClN_2_O_2_, 255.0900 (M + H)^+^; found, 255.0895.

### 4.2. Biology

#### 4.2.1. Cell Lines and Virus Strain

Human A549 (ATCC^®^ CCL-185™), HFF (ATCC^®^ SCRC-1041™) and HEK-293 (ATCC^®^ CRL-1573™) cell lines were obtained from the American Type Culture Collection (ATCC, Manassas, VA, USA). The 293 β5 stable cell line overexpressing the human β5 integrin subunit and the HAdV-5 *ts1* mutant were kindly provided by Dr. Glen Nemerow [45]. The cell lines were propagated in Dulbecco’s modified Eagle medium (DMEM, Life Technologies/Thermo Fisher, MA, USA) supplemented with 10% fetal bovine serum (FBS) (Omega Scientific, Tarzana, CA, USA), 10 mM HEPES, 100 units/mL penicillin, 4 mM L-glutamine, 100 μg/mL streptomycin, and 0.1 mM nonessential amino acids (complete DMEM). Wild-type HAdV-5 and HCMV (AD169) were from the ATCC. The HAdV-GFP used in this study is a replication-defective HAdV5 that contains a CMV promoter-driven enhanced green fluorescent protein (eGFP) reporter gene cassette in place of the E1/E3 regions [46]. HAdVs were propagated in 293 β5 cells and separated from the cellular lysate by cesium chloride density gradient centrifugation. We calculated virus concentration, in mg/mL, with the Bio-Rad Protein Assay (Bio-Rad Laboratories, CA, USA) and converted it to virus particles/mL (vp/mL) using 4 × 10^12^ vp/mg.

#### 4.2.2. Plaque Assay

Compounds were measured at concentrations of 10 μM and in a dose−response assay ranging from 10 to 0.3 μM using low MOI infections (0.06 vp/cell) in a plaque assay. In brief, 293 β5 cells were seeded in 6-well plates at a density of 4 × 10^5^ cells per well in duplicate for each condition. Cells were infected with HAdV5-GFP (0.06 vp/cell) and rocked for 2 h at 37 °C, when cells reached 80%–90% confluency. When the incubation was finished, the inoculum was removed followed by washing the cells once with PBS. The cells were then carefully overlaid with 4 mL/well of equal parts of 1.6% (water/vol) Difco Agar Noble (Becton, Dickinson & Co., Sparks, MD, USA) and 2× EMEM (Minimum Essential Medium Eagle, BioWhittaker, MD, USA) supplemented with 2×penicillin/streptomycin, 2× *L*-glutamine, and 10% FBS. The compounds were also added to the mixture in concentrations ranging from 10 to 0.3 μM. After incubation for 7 days at 37 °C, we scanned plates with a Typhoon FLA 9000 imager (GE Healthcare Life Sciences, MA, USA) and quantified plaques with ImageJ [47].

#### 4.2.3. Cytotoxicity Assay

The cytotoxicity of these derivatives was tested by commercial kit AlamarBlue^®^ (Ref. DAL1025, Invitrogen, MA, USA). A549 cells at a density of 5 × 10^3^ cells per well in 96-well plates were seeded. Decreasing concentrations of each derivative (200 μM, 150 μM, 100 μM, 80 μM, 60 μM, 40 μM, 30 μM, 20 μM, 10 μM, 5 μM, 2.5 μM, 0 μM) were diluted in 100 μL of Dulbecco’s Modified Eagle Medium (DMEM). Cells were then incubated at 37 °C for 48 h according to the kit protocol. The cytotoxic concentration 50 (CC_50_) value was obtained by using the statistical package GraphPad Prism. This assay was performed in duplicate.

#### 4.2.4. Virus Yield Reduction

A549 cells (1.5 × 10^5^ cells/well in a 24-well plate) were incubated for 24 h in 500 μL of complete DMEM and then infected with wild-type HAdV5 (100 vp/cell) when more than 90% of confluency were observed. Infected cells were incubated for 48 h at 37 °C in 500 μL of complete DMEM containing niclosamide (3) at 5 μM and derivative 16 at 10-fold IC_50_ concentration obtained in the plaque assay or the same volume of DMSO (positive control). After 48 h, cells were harvested and subjected to three rounds of freeze/thaw. Serial dilutions of clarified lysates were titrated on A549 cells (3 × 10^4^ cells/well), and we calculated TCID_50_ values using an endpoint dilution method [48].

#### 4.2.5. DNA Quantification by Real-Time PCR

A549 cells (1.5 × 10^5^ cells/well in a 24-well plate) were incubated for 24 h in 500 μL of complete DMEM and then infected with wild-type HAdV5 (100 vp/cell) when more than 90% of confluency were observed. Infected cells were incubated 24 h at 37 °C in 500 μL of complete DMEM containing niclosamide (3) at 5 μM concentration and compound **16** at 10-fold its IC_50_ concentration in the plaque assay or the same volume of DMSO (positive control). All samples were carried out in duplicate. After incubation for 24 h at 37 °C, DNA was purified from the cell lysate with the E.Z.N.A.^®^ Tissue DNA Kit (Omega Bio-tek, Norcross, GA, USA) following the manufacturer’s instructions. TaqMan primers and probes for a common region of the HAdV5 hexon were designed with the GenScript Real-Time PCR (TaqMan) Primer Design software (GenScript, Leiden, Netherlands). Oligonucleotides sequences were: AQ1: 5′-GCC ACG GTG GGG TTT CTA AAC TT-3′; AQ2: 5′-GCC CCA GTG GTC TTA CAT GCA CAT-3′; Probe: 6-FAM-5′-TGC ACC AGA CCC GGG CTC AGG TAC TCC GA-3′-TAMRA. Real-time PCR mixtures contained 9.5 μL of the purified DNA, AQ1 and AQ2 at a concentration of 200 nM each and Probe at a concentration of 50 nM in a total volume of 25 μL. The PCR cycling protocol was 95 °C for 3 min followed by 40 cycles of 95 °C for 10 s and 60 °C for 30 s. Human glyceraldehyde-3-phosphate dehydrogenase (GAPDH) gene was applied as internal control. Oligonucleotides sequences for GAPDH and conditions were previously reported by Rivera et al. [49].

For quantification, gene fragments from hexon and GAPDH were cloned into the pGEM-T Easy vector (Promega, MA, USA), and known concentrations of template were used to produce a standard curve in parallel for each experiment. All assays were done in thermal cycler LightCycler^®^ 96 System (Roche, Basel, Switzerland).

#### 4.2.6. Nuclear-Associated HAdV Genomes

The nuclear delivery of HAdV genomes was evaluated by real-time PCR following nuclear isolation from infected cells. 1 × 10^6^ A549 cells in 6-well plates were infected with wild-type HAdV5 at MOI 2000 vp/cell in the presence of 10-fold IC_50_ concentration obtained in the plaque assay for compound **16**, niclosamide (3) at 5 μM or the same volume of DMSO for positive control. Forty-five minutes after infection, A549 cells were trypsinized, collected, and then washed twice with PBS. After that, we separated cytoplasmic and nuclear fractions using a hypotonic buffer solution and NP-40 detergent and resuspended the cell pellet in 500 μL of 1 × hypotonic buffer (20 mM Tris-HCl pH 7.4, 10 mM NaCl, 3 mM MgCl_2_). After incubation for 15 min at 4 °C, 25 μL of NP-40 was added and the samples were vortexed. The homogenates were centrifuged for 10 min at 835g and 4 °C. After the removal of the cytoplasmic fraction (supernatant), HAdV DNA was separated from the nuclear fraction (pellet) and from the cytoplasmic fraction using the E.Z.N.A.^®^ Tissue DNA Kit (Omega Bio-tek, Norcross, GA, USA).

#### 4.2.7. Time of Addition Assay

The anti-HAdV effects of compounds 3 and 16 at different points were evaluated in a time-curve assay using 293 β5 cells (3 × 10^5^ cells/well in corning black wall, clear bottom 96-well plates) that were infected with HAdV5-GFP (2000 vp/cell) in the presence of 5 μM of compound **3** and 10-fold IC_50_ concentration obtained in the plaque assay for derivative 16. Parallel samples of HAdV-5 were incubated with or without the selected compounds on ice for 1 h. Virus was then added to 293 β5 cells and incubated at 37 °C. Compounds 3 and 16 were added at the indicated time points before or during this incubation. After a total of 2 h at 37 °C, cells were incubated for an additional 48 h at 37 °C and 5% CO_2_ before analyzing GFP expression using the Typhoon 9410 imager (GE Healthcare Life Sciences, MA, USA) as above.

#### 4.2.8. HAdV-Mediated Endosome Disruption

A549 cells (~20,000 cells/well) were incubated in DMEM without cysteine or methionine supplemented with 10% dialyzed FBS [DMEM (−)] in black 96-well plates for 1 h before infection. Threefold serial dilutions (0.45 ng to 1000 ng) of HAdV5, or HAdV-5 *ts1* were preincubated with cells in the presence of 5 µM niclosamide (3), 10-fold the IC_50_ concentration obtained in the plaque assay for compound **16**, or the same volume of DMSO (negative control) for one hour. The medium was then removed and replaced with 50 μL DMEM (−) containing 0.1 mg/mL α-sarcin (Santa Cruz Biotechnology, Dallas, TX, USA) and the virus and drug mixtures. After 2 h at 37 °C, the Click-iT HPG Alexa Fluor 488 Protein Synthesis Assay Kits (Invitrogen, MA, USA) were used to analyze protein synthesis according to the manufacturer’s instructions. The incorporation of the amino acid analog of methionine L-homopropargylglycine (HPG) containing Alexa Fluor 488 azide was measured using a Typhoon 9410 imager (GE Healthcare Life Sciences, MA, USA) and calculated subtracting the background level of the control well containing L-homopropargylglycine (HPG) and α-sarcin but not virus (100% incorporation).

#### 4.2.9. Thermostability Assay

HAdV-GFP (37.5 ng) was incubated with compound **16** (50 µM) or the same volume of DMSO for 1 h in complete DMEM. Parallel samples were incubated for 10 min at the indicated temperatures, cooled to r.t., and added to A549 cells on ice to synchronize the infection. After the incubation, cells were washed twice with PBS 1x to remove the nonattached virus and the excess of compound **16** and DMSO. Cells were then incubated for 24 h at 37 °C and 5% CO_2_ before being visualized for GFP expression in an Olympus inverted microscope Model IX71 (Hamburg, Germany) and analyzed by capturing representative images of each condition using the CellSens Dimension platform (Olympus, Hamburg, Germany).

#### 4.2.10. Statistical Analyses

One-way ANOVA tests (Dunnet method) were performed using the GraphPad Prism 6. A *P* value under 0.05 was considered a statistical significance. We pointed out this statistical significance with asterisk in graphs, the numbers of which indicate the level of significance (* *p* ≤ 0.05, ** *p* ≤ 0.01, *** *p* ≤ 0.001, **** *p* ≤ 0.0001).

## Figures and Tables

**Table 1:** Inhibition of HAdV in plaque assay, cytotoxicity, and selectivity index for compounds 11–28.

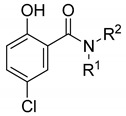						
Compound	R^1^	R^2^	Plaque Assay (10 μM) *^a^*		CC_50_ (μM) *^b^*	Selectivity Index (SI) *^c^*
			**(%) Inhibition**	**IC_50_ (μM)**		
**3**	NA		100 ± 0	0.6 ± 0.05	22.9 ± 9.8	38.2
**11**	H	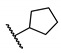	22.6 ± 7.4	NT *^d^*	NT	NT
**12**	H	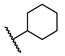	57.6 ± 0.2	NT	NT	NT
**13**	H	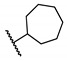	99.6 ± 0.2	4.7 ± 0.1	30.9 ± 6.3	6.6
**14**	H	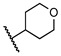	0.0 ± 0.0	NT	NT	NT
**15**	H	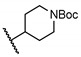	52.2 ± 7.1	NT	NT	NT
**16**	H	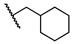	100 ± 0	0.78 ± 0.01	91.2 ± 18.4	116.9
**17**	H	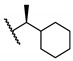	100 ± 0	0.51 ± 0.14	23.0 ± 1.3	45.1
**18**	H	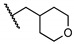	0.0 ± 0.0	NT	NT	NT
**19**	H	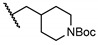	88.5 ± 1.9	NT	NT	NT
**20**	H	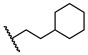	97.8 ± 1.7	1.0 ± 0.1	66.3 ± 7.9	64.3
**21**	H	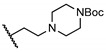	22.2 ± 13.2	NT	NT	NT
**22**	H	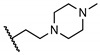	0.00 ± 0.00	NT	NT	NT
**23**	H	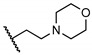	0.00 ± 0.00	NT	NT	NT
**24**	H	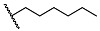	98.5 ± 2.1	3.73 ± 0.64	23.8 ± 3.1	6.4
**25**	H	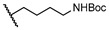	74.0 ± 5.7	NT	NT	NT
**26**	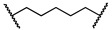		0.00 ± 0.00	NT	NT	NT
**27**	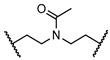		0.00 ± 0.00	NT	NT	NT
**28**	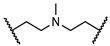		0.00 ± 0.00	NT	NT	NT

*^a^* Percentage of control HAdV5-GFP inhibition at 10 μM and inhibitory concentration 50% (IC_50_) at low MOI in a plaque assay using the 293β5 cell line. *^b^* Cytotoxic concentration 50% (CC_50_) using A549 cell line. *^c^* Selectivity index value was determined as the ratio of CC_50_ to IC_50_ in a plaque assay for each compound. *^d^* NT: not tested. The results represent means ± SD of triplicate samples from three independent experiments.

**Table 2:** Virus yield reduction for derivative 16.

Compound	Virus Yield Reduction (fold) *^a^*
niclosamide	82 ± 35
**16**	208 ± 108

*^a^* Fold reduction in virus yield as the ratio of particles produced in the presence of DMSO divided by the yield in the presence of niclosamide (3) at 5 μM and derivative 16 at 10-fold IC_50_ concentration obtained in the plaque assay. Virus yield reduction assay used A549 cell line and the MOI of HAdV was 100 vp/cell. The results represent means ± SD of triplicate samples from three independent experiments.

**Table 3:** IC_50_ values in a plaque assay for each derivative and its combination. Combination index value was calculated with CalcuSyn software.

	Combinatory Index (CI) Values in Plaque Assay			
Combination (Ratio)	ED_50_	ED_75_	ED_90_	*r*
4 and 16 (1:4.3)	0.26	0.38	0.56	0.87
5 and 16 (1:2.9)	1.09	1.08	1.14	0.92
6 and 16 (1:1.73)	0.40	0.27	0.19	0.94
4, 5, and 16 (1:1.5:4.3)	0.33	0.26	0.22	0.94
5, 6, and 16 (1:1.7:2.9)	2.01	1.17	0.72	0.95

## Data Availability

The data presented in this study are available on request from the corresponding author.

## Supplementary Materials

Supplementary Materials can be found at https://www.mdpi.com/1422-0067/22/4/1617/s1, Copies of ^1^H and ^13^C NMR spectra.

## Abbreviations

allo-HSCT	allogenic hematopoietic stem cell transplant
BCV	brincidofovir
CAP	community-acquired pneumonia
CC_50_	half-maximal cytotoxic concentration
DCM	dichloromethane
DMAP	4-(dimethylamino)pyridine
DMSO	dimethyl sulfoxide
EDCI	1-(3-dimethylaminopropyl)-3-ethylcarbodiimide hydrochloride
EtOAc	ethyl acetate
HAdVs	human adenoviruses
HCMV	human cytomegalovirus
HPG	L-homopropargylglycine
HPLC	high-performance liquid chromatography
HRMS	high-resolution mass spectrometry
IC_50_	half-maximal inhibitory concentration
p.i.	postinfection
SAR	structure–activity relationship
SOT	solid-organ transplant
TLC	thin layer chromatography
TMS	tetramethylsilane
UV	ultraviolet
