# Supplementary material for: Discovery of a Small Molecule Inhibitor of Human Adenovirus Capable of Preventing Escape from the Endosome
Source: Int J Mol Sci. 2021 Feb 5;22(4):1617. doi: 10.3390/ijms22041617 (PMC7915867; doi:10.3390/ijms22041617)
Supplement: Supplementary file 1 [file ijms-22-01617-s001.pdf]

Supporting Information

Discovery of a small molecule inhibitor of human adenovirus capable of preventing escape from the endosome

Jimin Xu [1,†], Judith Berastegui-Cabrera [2,†], Marta Carretero-Ledesma [2], Haiying Chen [1], Yu Xue [1], Eric A. Wold [1], Jerónimo Pachón-Díaz [2,3], Jia Zhou [1,*] and Javier Sánchez-Céspedes [2,*]

[1] Chemical Biology Program, Department of Pharmacology and Toxicology, University of Texas Medical Branch, Galveston, Texas 77555, United States

[2] Unit of Infectious Diseases, Microbiology and Preventive Medicine, Institute of Biomedicine of Seville (IBiS), University Hospital Virgen del Rocío/CSIC/University of Seville, E41013 Seville, Spain

[3] Department of Medicine, University of Seville, E-41009 Seville, Spain

Table of Contents

Copies of ^{1}H and ^{13}C NMR spectra……………………………………………………..S2-S19

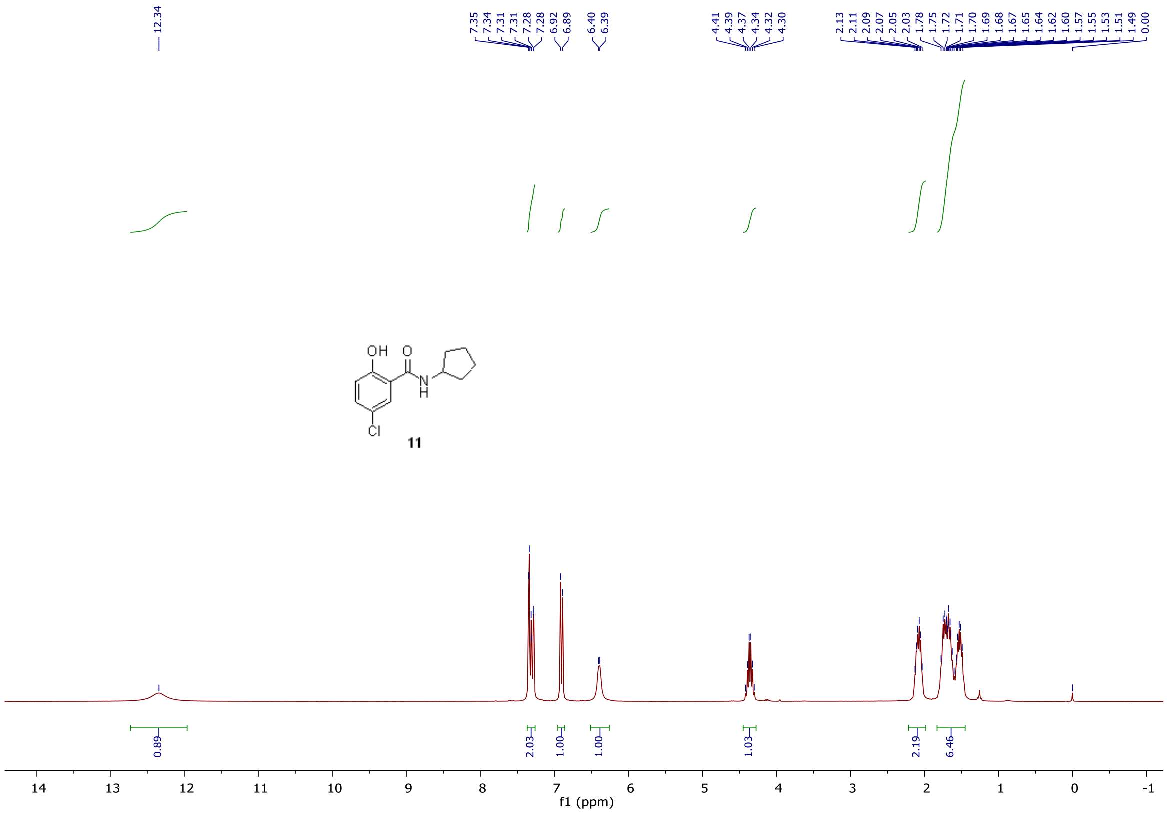
12.34
7.35 7.34 7.31 7.31 7.28 7.28 6.92 6.89 6.40 6.39
4.41 4.39 4.37 4.34 4.32 4.30
2.13 2.11 2.09 2.07 2.05 2.03 1.78 1.75 1.72 1.71 1.70 1.69 1.68 1.67 1.65 1.64 1.62 1.60 1.57 1.55 1.53 1.51 1.49 0.00
OH
O
N
H
Cl
11
0.89
2.03
1.00
1.00
1.03
2.19
6.46
f1 (ppm)

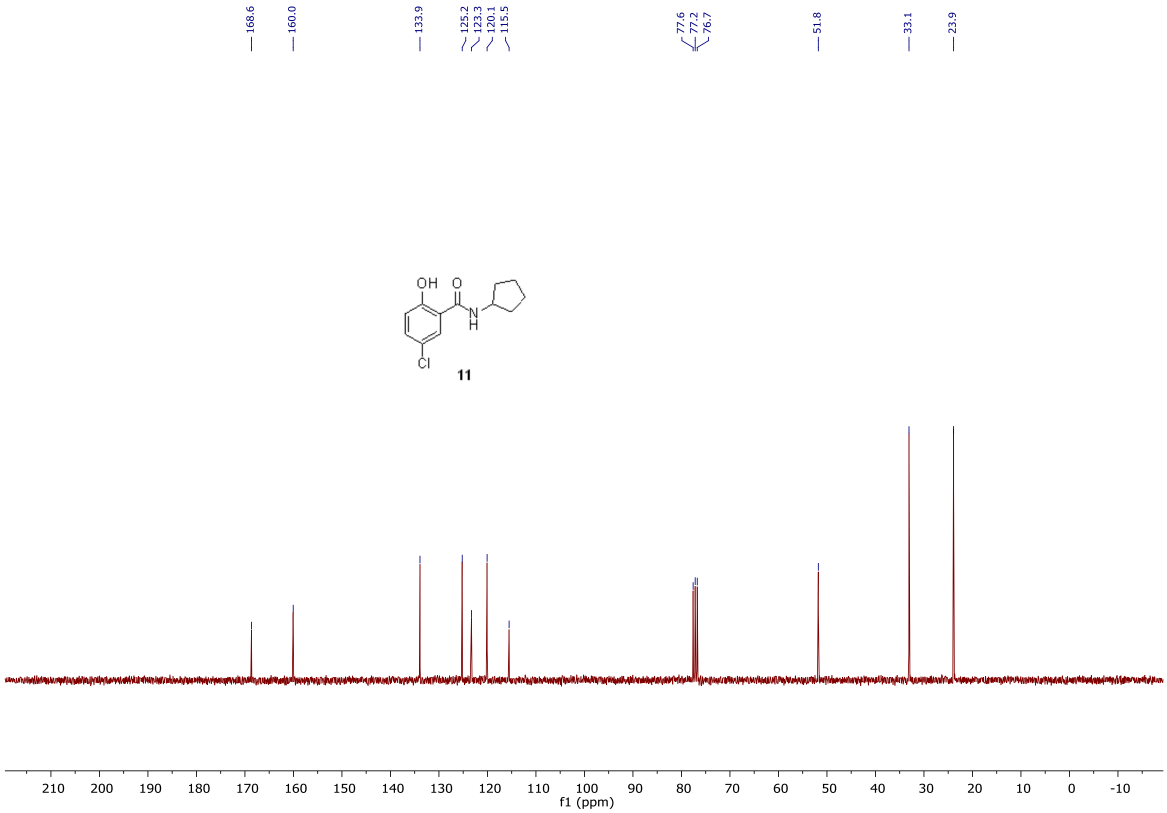
168.6
160.0
133.9
125.2 123.3 120.1 115.5
77.6 77.2 76.7
51.8
33.1
23.9
OH
O
N
H
Cl
11
f1 (ppm)

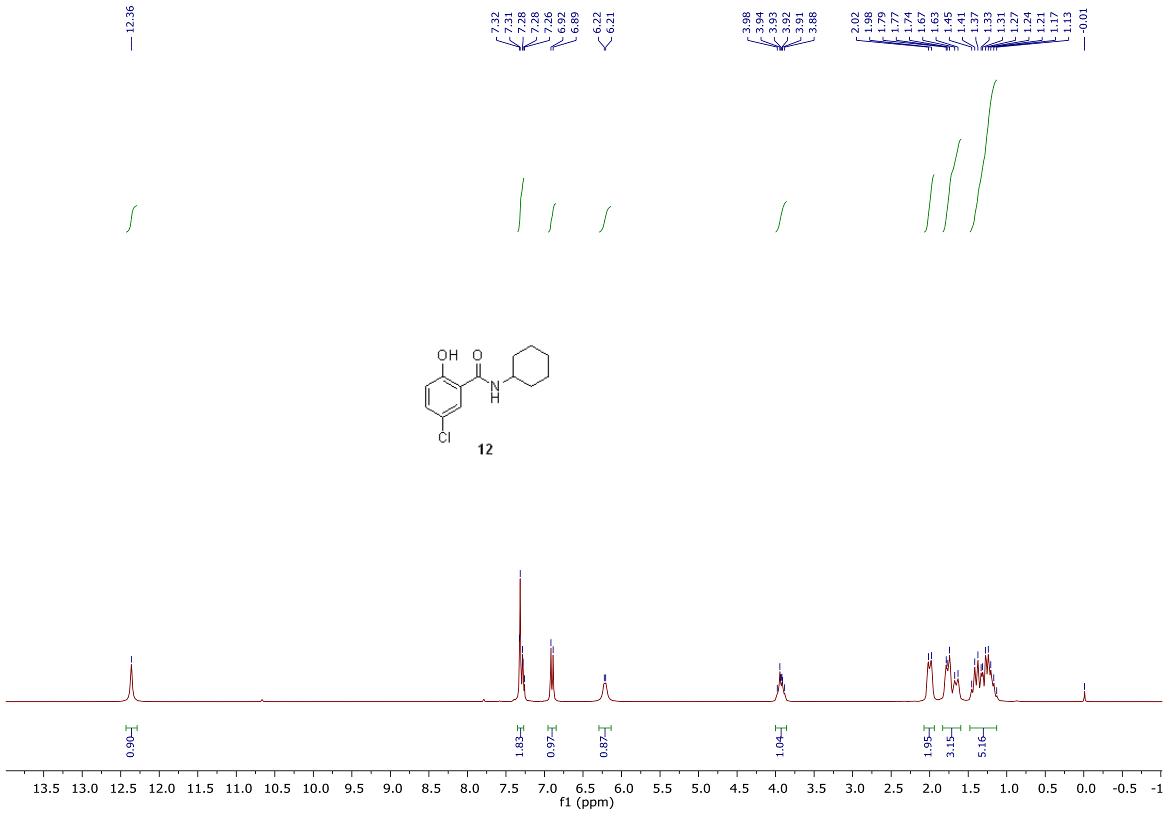
12.36
7.32 7.31 7.28 7.28 7.26 6.92 6.89
6.22 6.21
3.98 3.94 3.93 3.92 3.91 3.88
2.02 1.98 1.79 1.77 1.74 1.67 1.63 1.45 1.41 1.37 1.33 1.31 1.27 1.24 1.21 1.17 1.13
-0.01
OH
O
N
H
Cl
12
0.90
1.83
0.97
0.87
1.04
1.95
3.15
5.16
f1 (ppm)

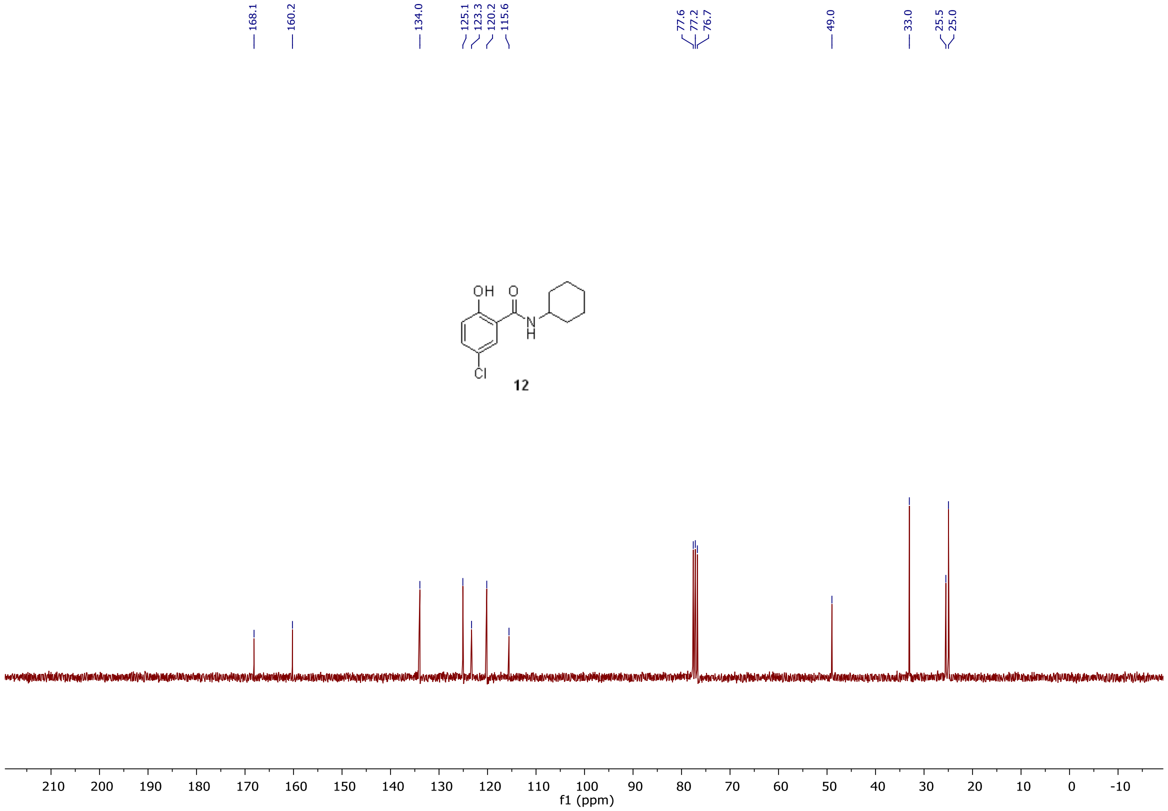
168.1
160.2
134.0
125.1 123.3 120.2 115.6
77.6 77.2 76.7
49.0
33.0
25.5 25.0
OH
O
N
H
Cl
12
f1 (ppm)

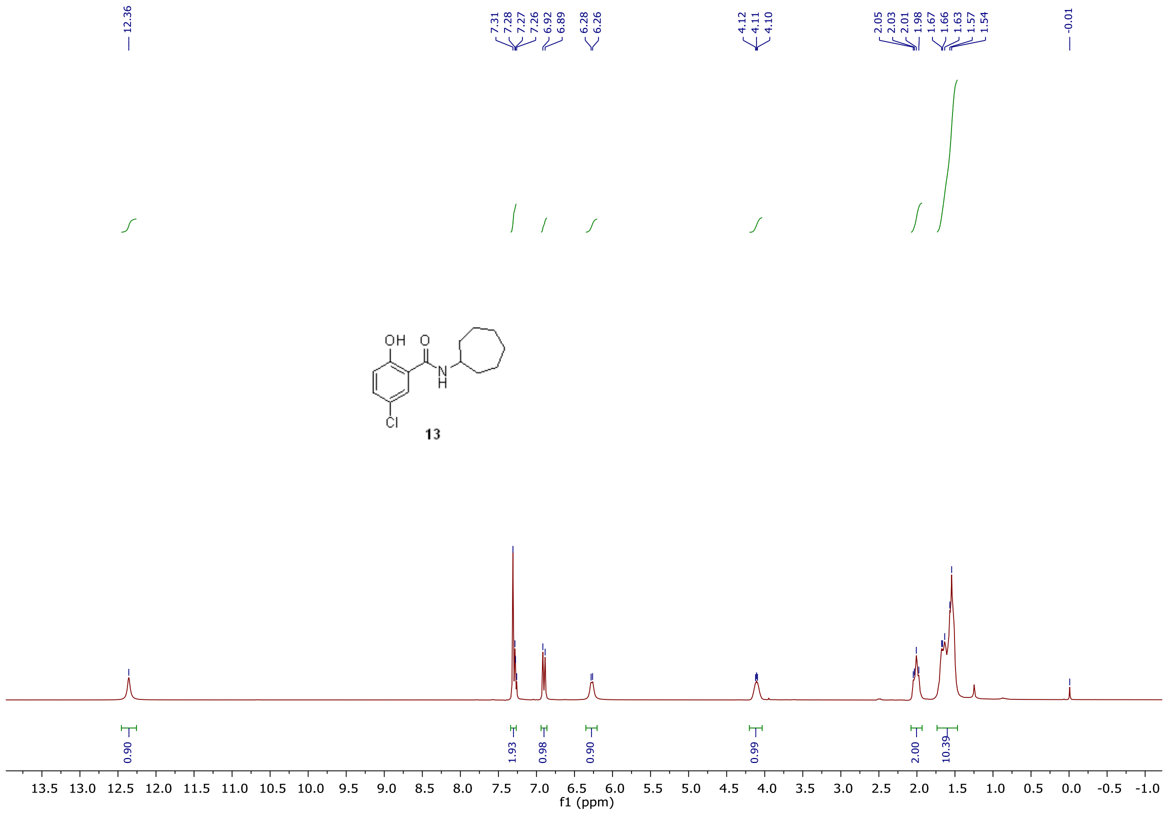
12.36
7.31
7.28
7.27
7.26
6.92
6.89
6.28
6.26
4.12
4.11
4.10
2.05
2.03
2.01
1.98
1.67
1.66
1.63
1.57
1.54
-0.01
OH
O
N
H
Cl
13
0.90
1.93
0.98
0.90
0.99
2.00
10.39
13.5 13.0 12.5 12.0 11.5 11.0 10.5 10.0 9.5 9.0 8.5 8.0 7.5 7.0 6.5 6.0 5.5 5.0 4.5 4.0 3.5 3.0 2.5 2.0 1.5 1.0 0.5 0.0 -0.5 -1.0
f1 (ppm)

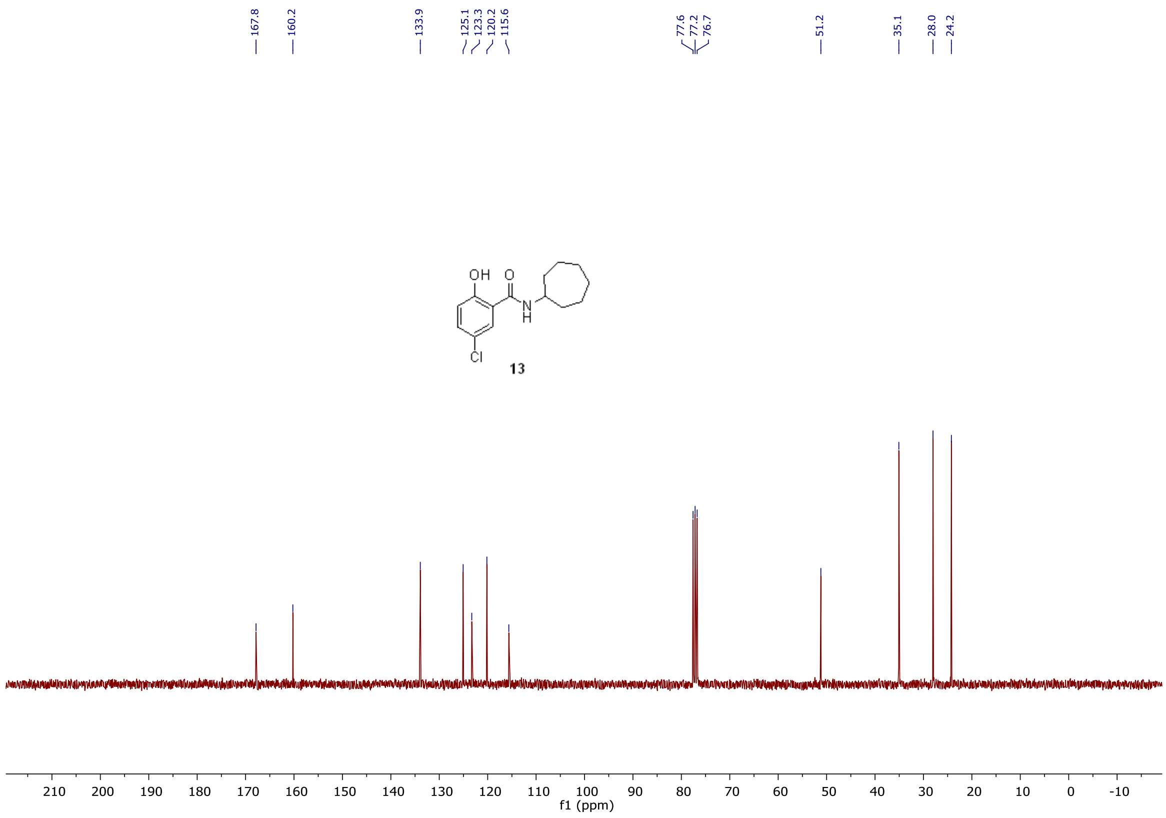
167.8
160.2
133.9
125.1
123.3
120.2
115.6
77.6
77.2
76.7
51.2
35.1
28.0
24.2
OH
O
N
H
Cl
13
210 200 190 180 170 160 150 140 130 120 110 100 90 80 70 60 50 40 30 20 10 0 -10
f1 (ppm)

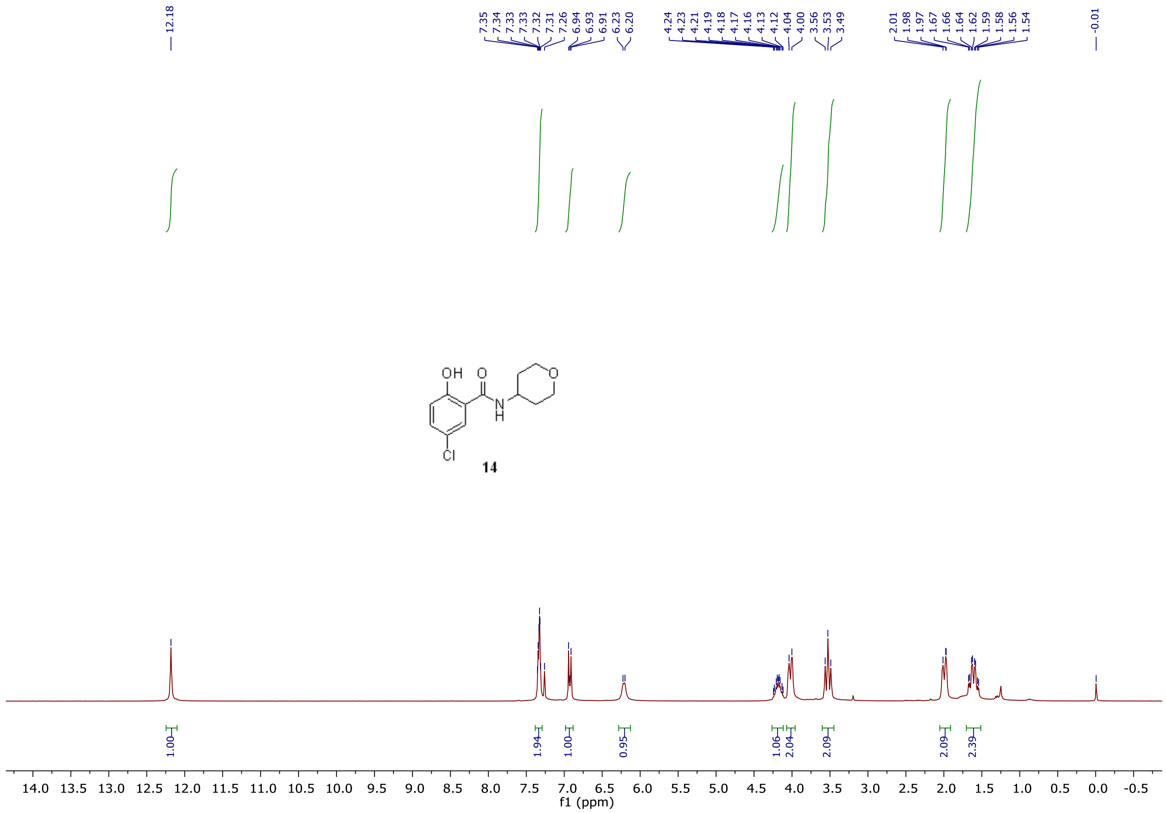
12.18
7.35 7.34 7.33 7.33 7.32 7.31 7.26 6.94 6.93 6.91 6.23 6.20
4.24 4.23 4.21 4.19 4.18 4.17 4.16 4.13 4.12 4.04 4.00 3.56 3.53 3.49
2.01 1.98 1.97 1.67 1.66 1.64 1.62 1.59 1.58 1.56 1.54
-0.01
OH
O
N
H
O
Cl
14
1.00
1.94
1.00
0.95
1.06
2.04
2.09
2.09
2.39
f1 (ppm)

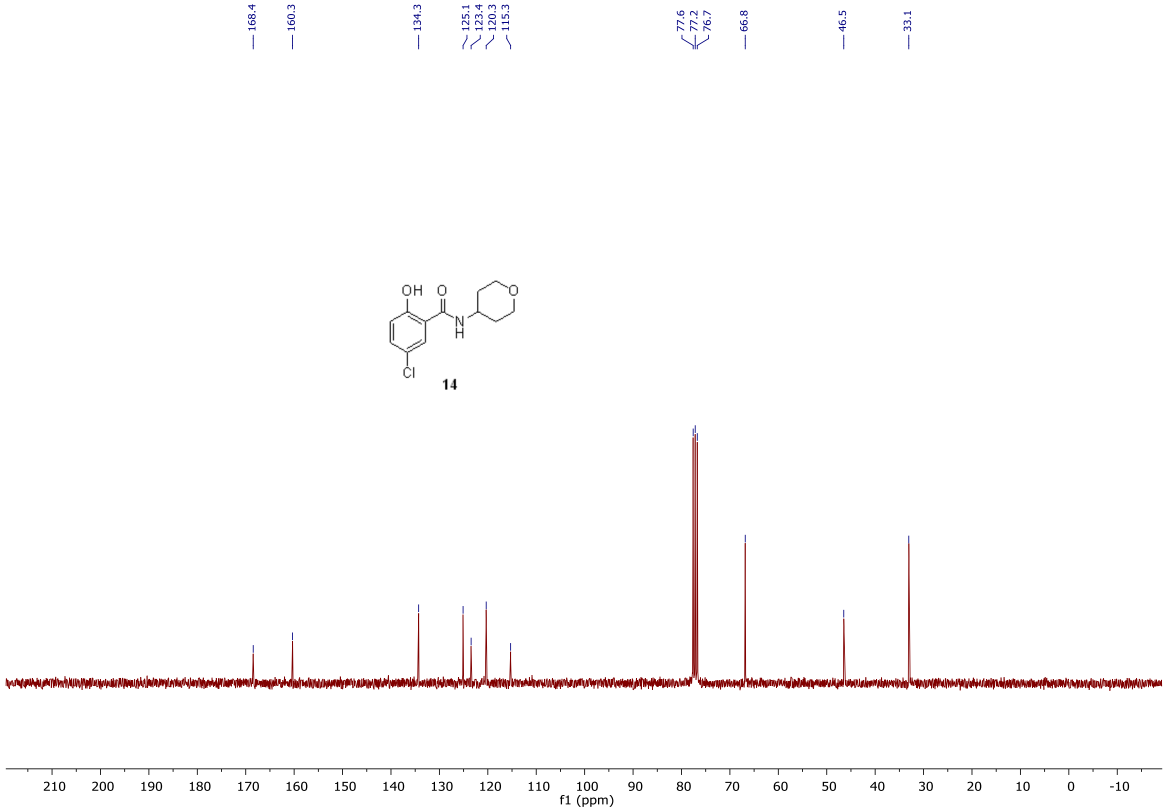
168.4
160.3
134.3
125.1 123.4 120.3 115.3
77.6 77.2 76.7
66.8
46.5
33.1
OH
O
N
H
O
Cl
14
f1 (ppm)

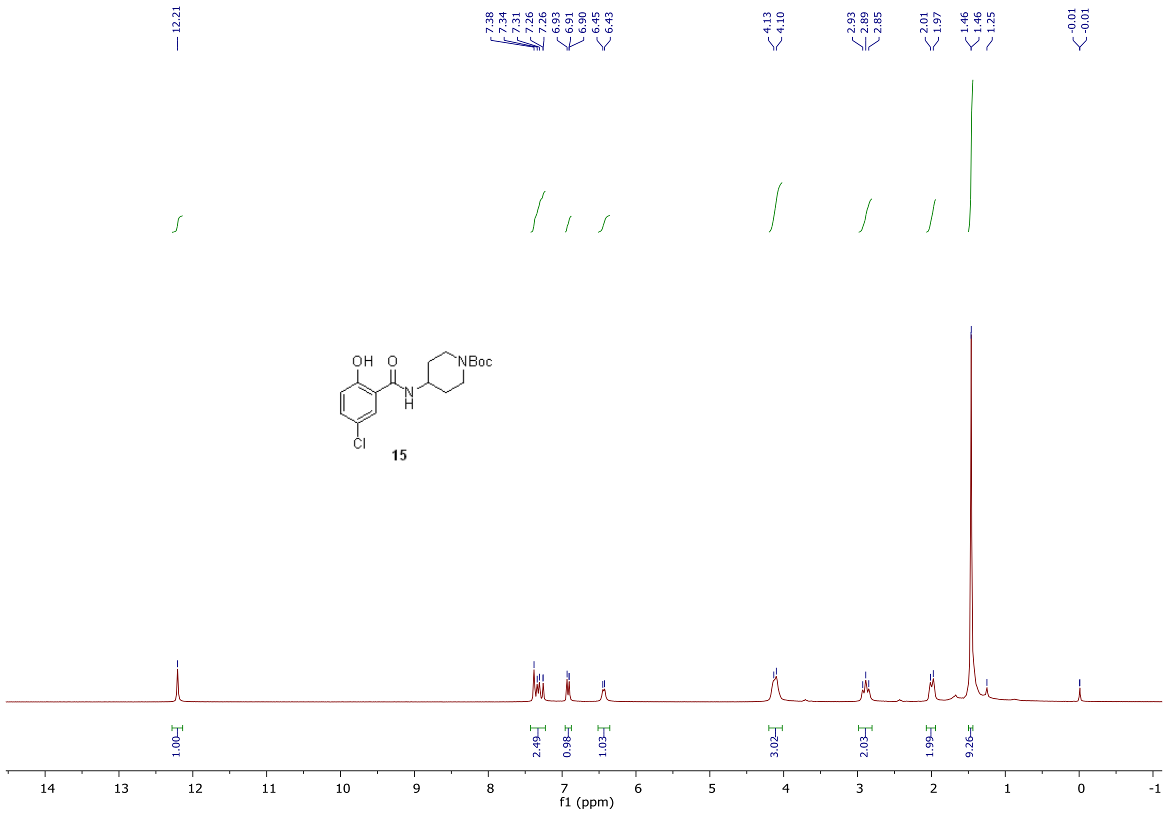
12.21
7.38 7.34 7.31 7.26 7.26 6.93 6.91 6.90 6.45 6.43
4.13 4.10
2.93 2.89 2.85
2.01 1.97 1.46 1.46 1.25
-0.01 -0.01
OH O
NBoc
N
H
Cl
15
1.00 2.49 0.98 1.03 3.02 2.03 1.99 9.26
14 13 12 11 10 9 8 7 6 5 4 3 2 1 0 -1
f1 (ppm)

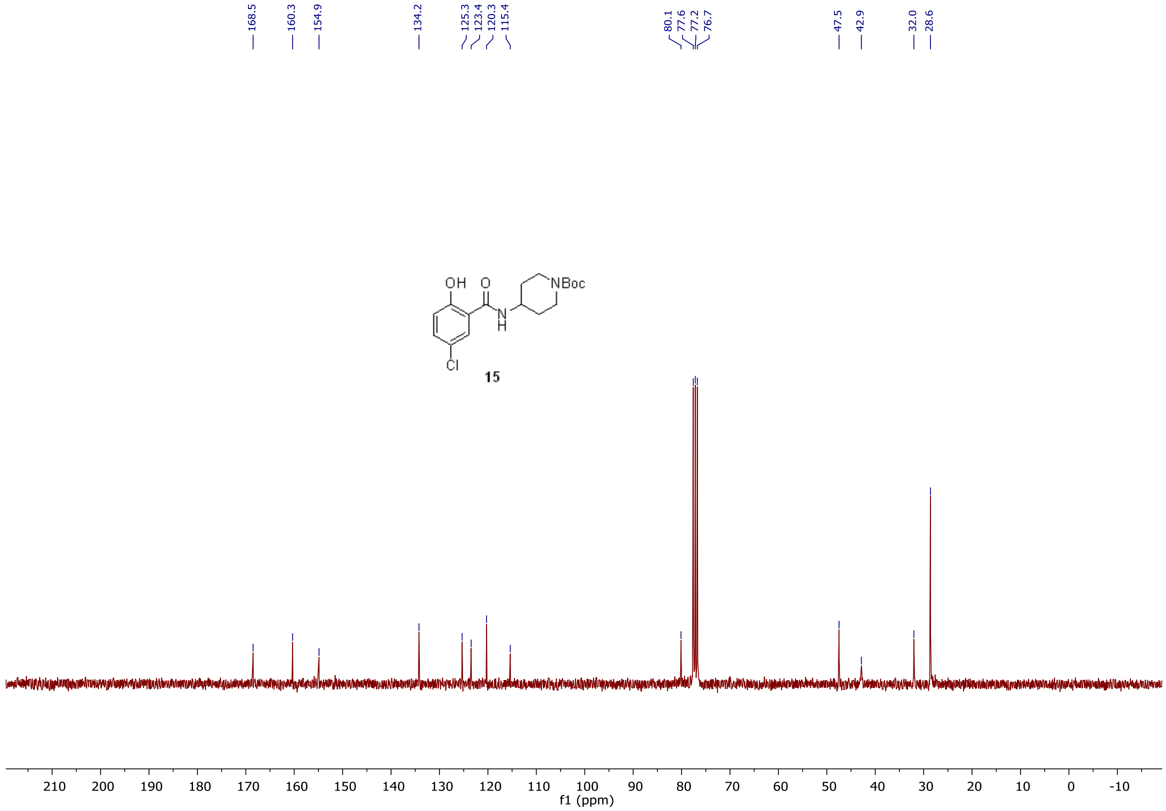
168.5 160.3 154.9 134.2 125.3 123.4 120.3 115.4 80.1 77.6 77.2 76.7 47.5 42.9 32.0 28.6
OH O
NBoc
N
H
Cl
15
210 200 190 180 170 160 150 140 130 120 110 100 90 80 70 60 50 40 30 20 10 0 -10
f1 (ppm)

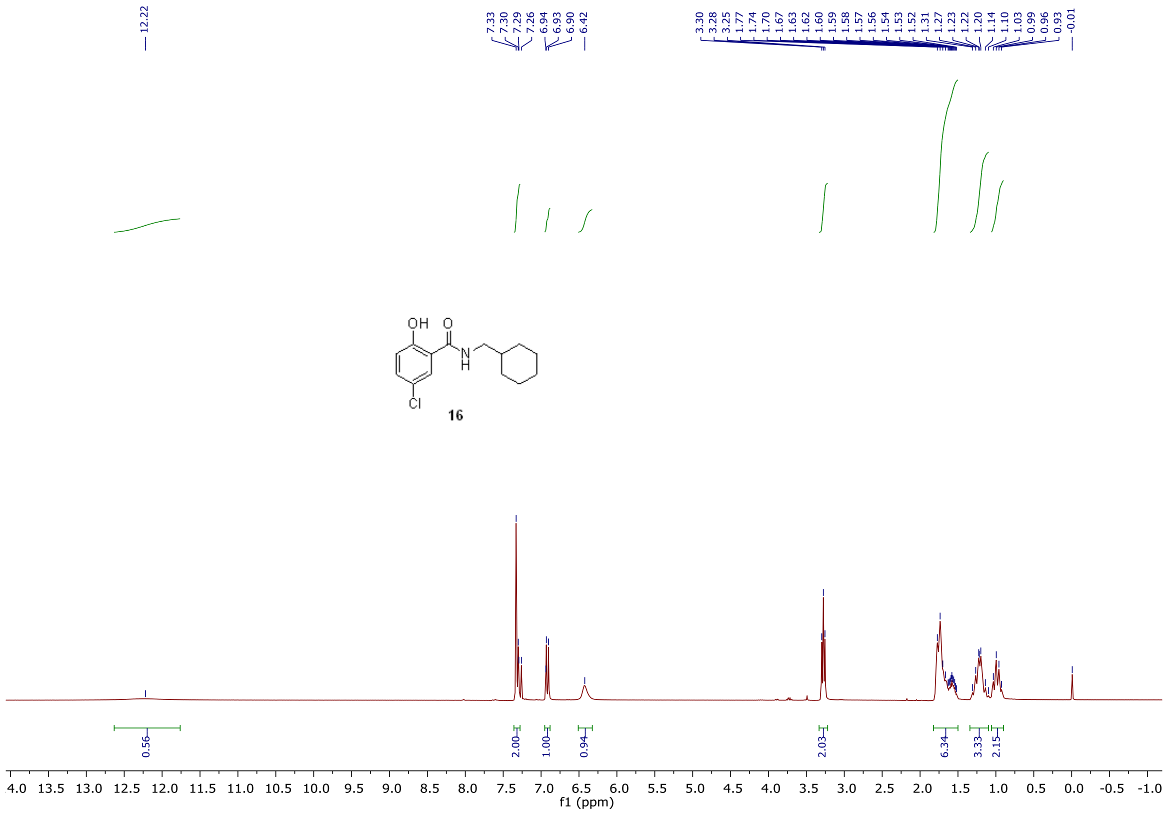
12.22
7.33
7.30
7.29
7.26
6.94
6.93
6.90
6.42
3.30
3.28
3.25
1.77
1.74
1.70
1.67
1.63
1.62
1.60
1.59
1.58
1.57
1.56
1.54
1.53
1.52
1.31
1.27
1.23
1.22
1.20
1.14
1.10
1.03
0.99
0.96
0.93
-0.01
OH
O
N
H
Cl
16
0.56
2.00
1.00
0.94
2.03
6.34
3.33
2.15
f1 (ppm)

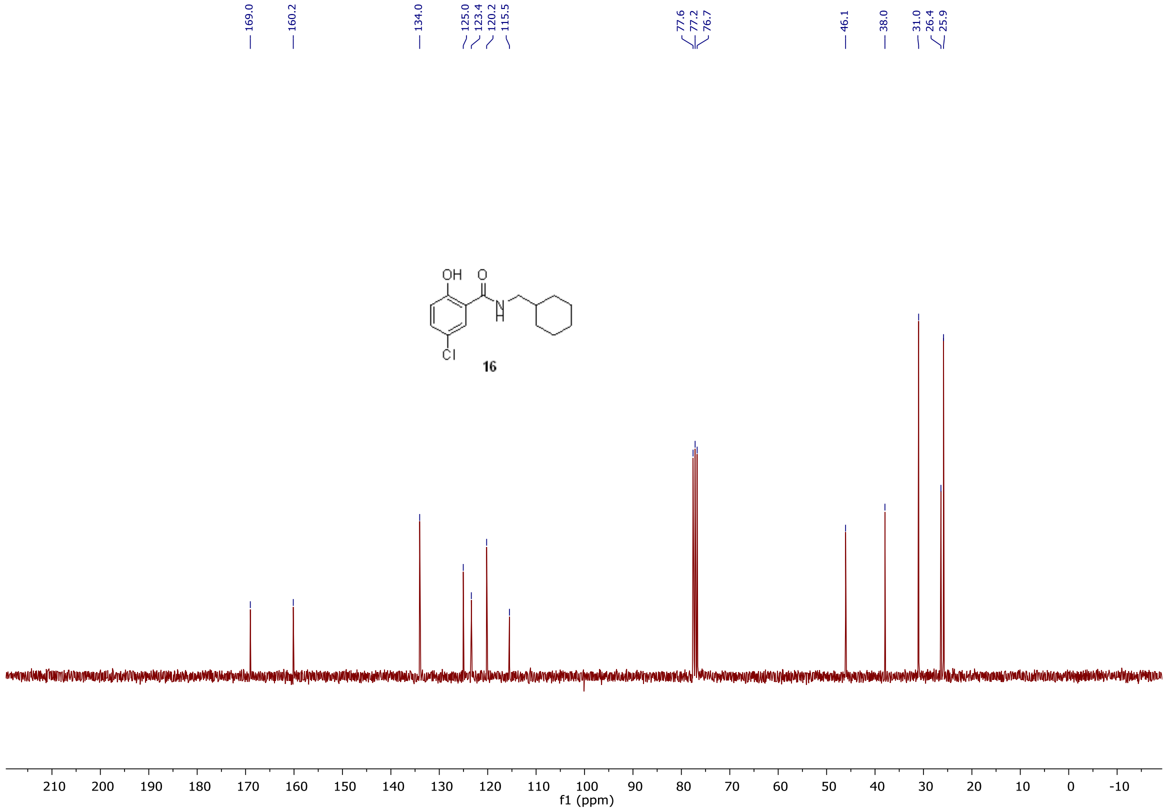
169.0
160.2
134.0
125.0
123.4
120.2
115.5
77.6
77.2
76.7
46.1
38.0
31.0
26.4
25.9
OH
O
N
H
Cl
16
f1 (ppm)

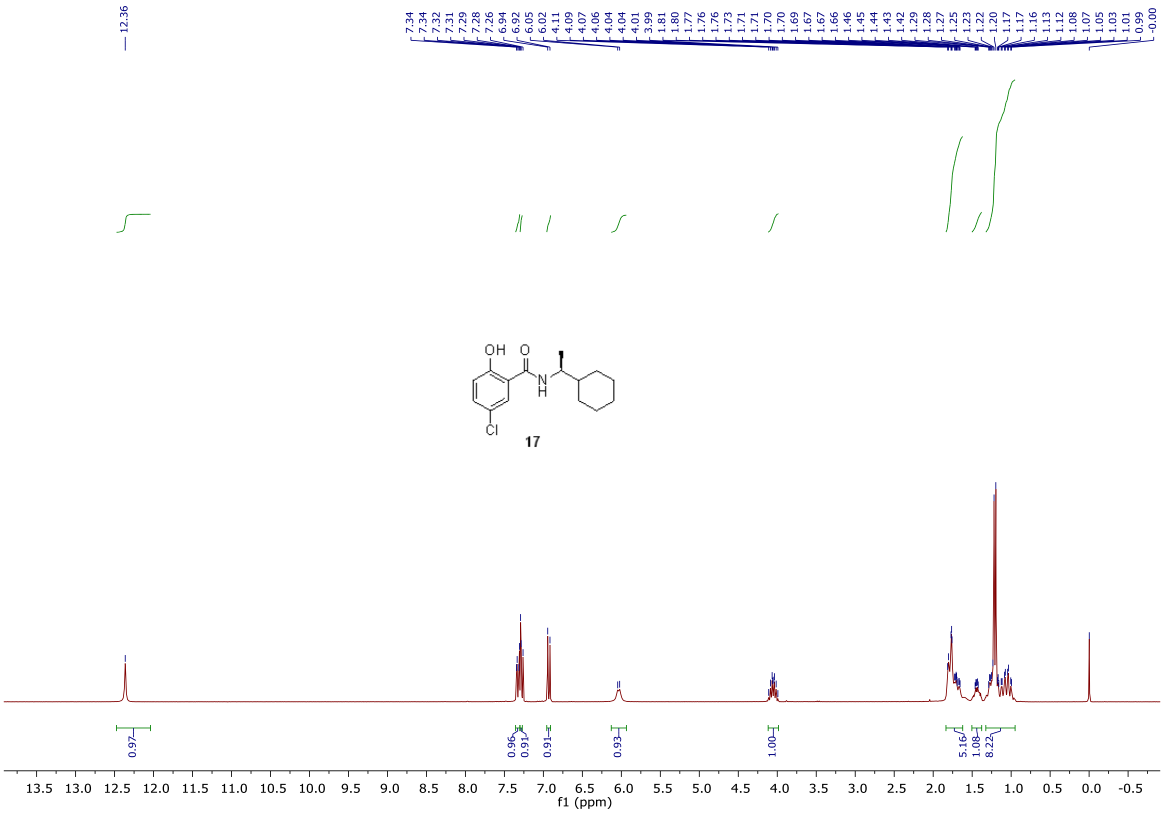
12.36
7.34 7.34 7.32 7.31 7.29 7.28 7.26 6.94 6.92 6.05 6.02 4.11 4.09 4.07 4.06 4.04 4.04 4.01 3.99 1.81 1.80 1.77 1.76 1.76 1.73 1.71 1.71 1.70 1.70 1.69 1.67 1.67 1.66 1.46 1.45 1.44 1.43 1.42 1.29 1.28 1.27 1.25 1.23 1.22 1.20 1.17 1.17 1.16 1.13 1.12 1.08 1.07 1.05 1.03 1.01 0.99 -0.00
OH
O
N
H
Cl
17
0.97
0.96
0.91
0.91
0.93
1.00
5.16
1.08
8.22
13.5 13.0 12.5 12.0 11.5 11.0 10.5 10.0 9.5 9.0 8.5 8.0 7.5 7.0 6.5 6.0 5.5 5.0 4.5 4.0 3.5 3.0 2.5 2.0 1.5 1.0 0.5 0.0 -0.5
f1 (ppm)

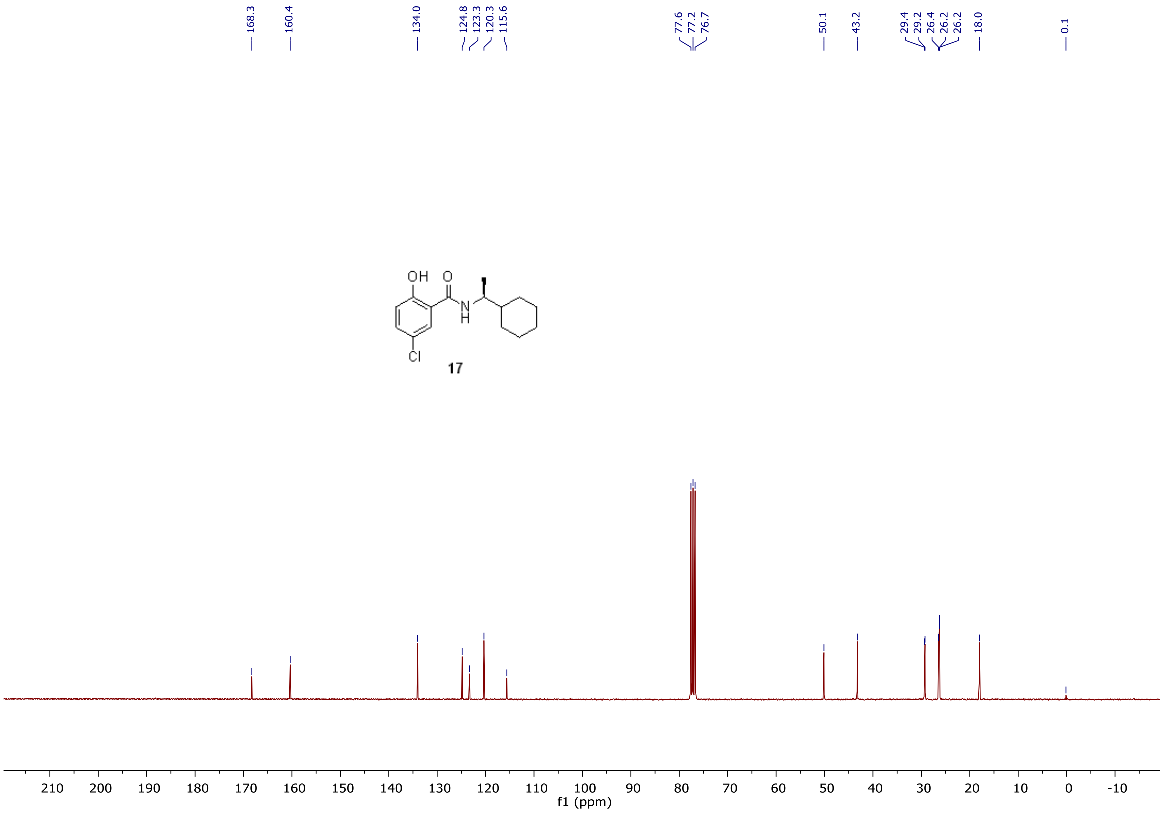
168.3
160.4
134.0
124.8 123.3 120.3 115.6
77.6 77.2 76.7
50.1
43.2
29.4 29.2 26.4 26.2 26.2
18.0
0.1
OH
O
N
H
Cl
17
210 200 190 180 170 160 150 140 130 120 110 100 90 80 70 60 50 40 30 20 10 0 -10
f1 (ppm)

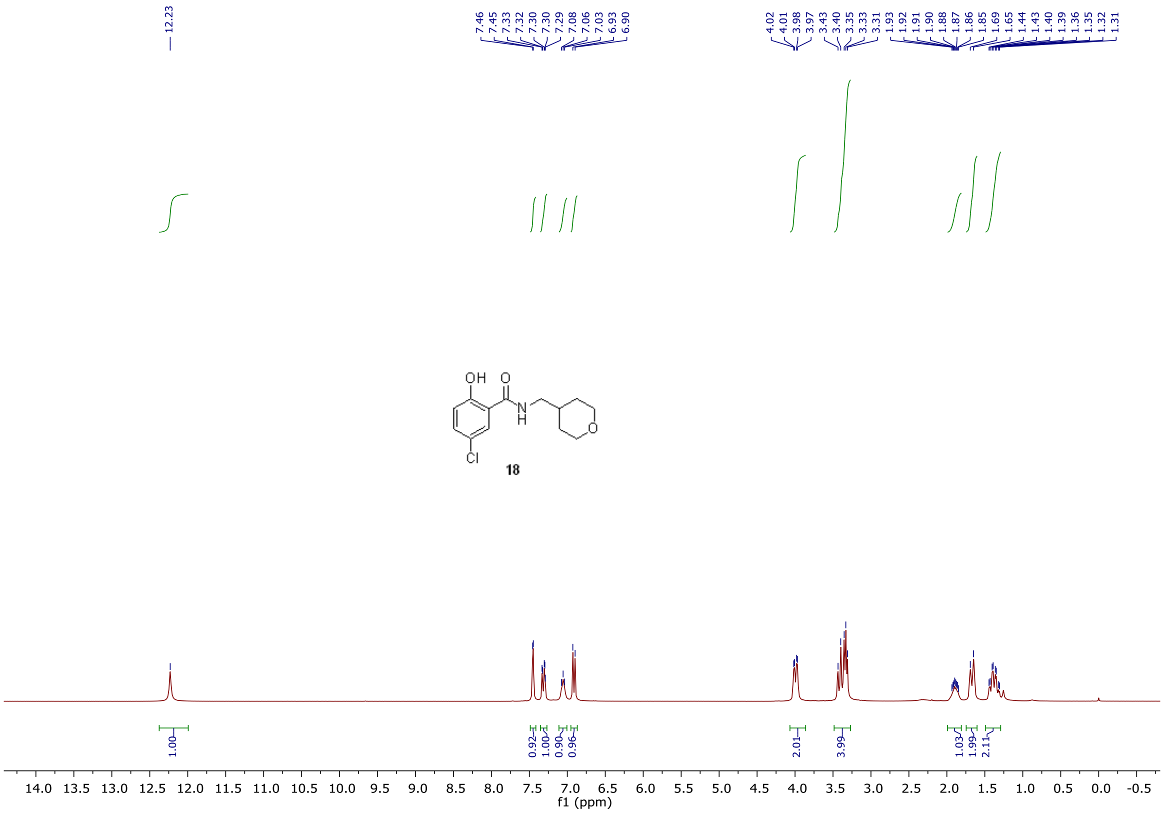
12.23
7.46
7.45
7.33
7.32
7.30
7.30
7.29
7.08
7.06
7.03
6.93
6.90
4.02
4.01
3.98
3.97
3.43
3.40
3.35
3.33
3.31
1.93
1.92
1.91
1.90
1.88
1.87
1.86
1.85
1.69
1.65
1.44
1.43
1.40
1.39
1.36
1.35
1.32
1.31
OH
O
N
H
O
Cl
18
1.00
0.92
1.00
0.90
0.96
2.01
3.99
1.03
1.99
2.11
14.0 13.5 13.0 12.5 12.0 11.5 11.0 10.5 10.0 9.5 9.0 8.5 8.0 7.5 7.0 6.5 6.0 5.5 5.0 4.5 4.0 3.5 3.0 2.5 2.0 1.5 1.0 0.5 0.0 -0.5
f1 (ppm)

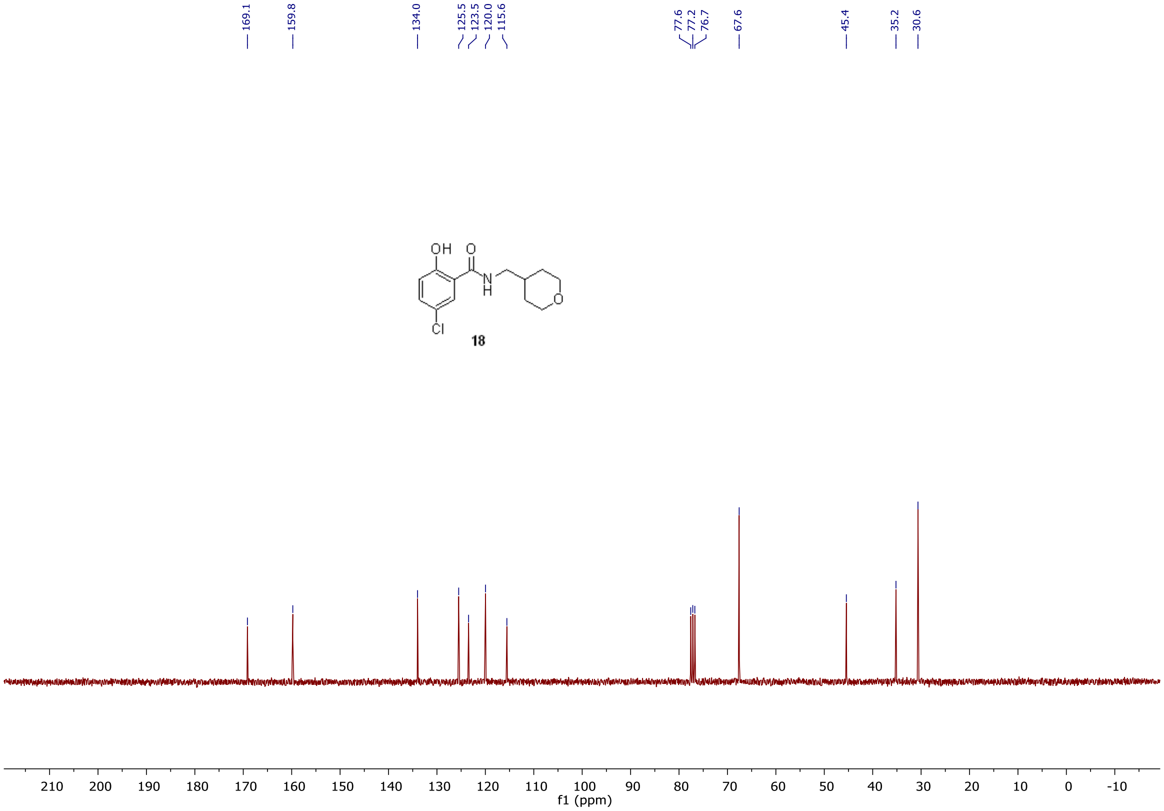
169.1
159.8
134.0
125.5
123.5
120.0
115.6
77.6
77.2
76.7
67.6
45.4
35.2
30.6
OH
O
N
H
O
Cl
18
210 200 190 180 170 160 150 140 130 120 110 100 90 80 70 60 50 40 30 20 10 0 -10
f1 (ppm)

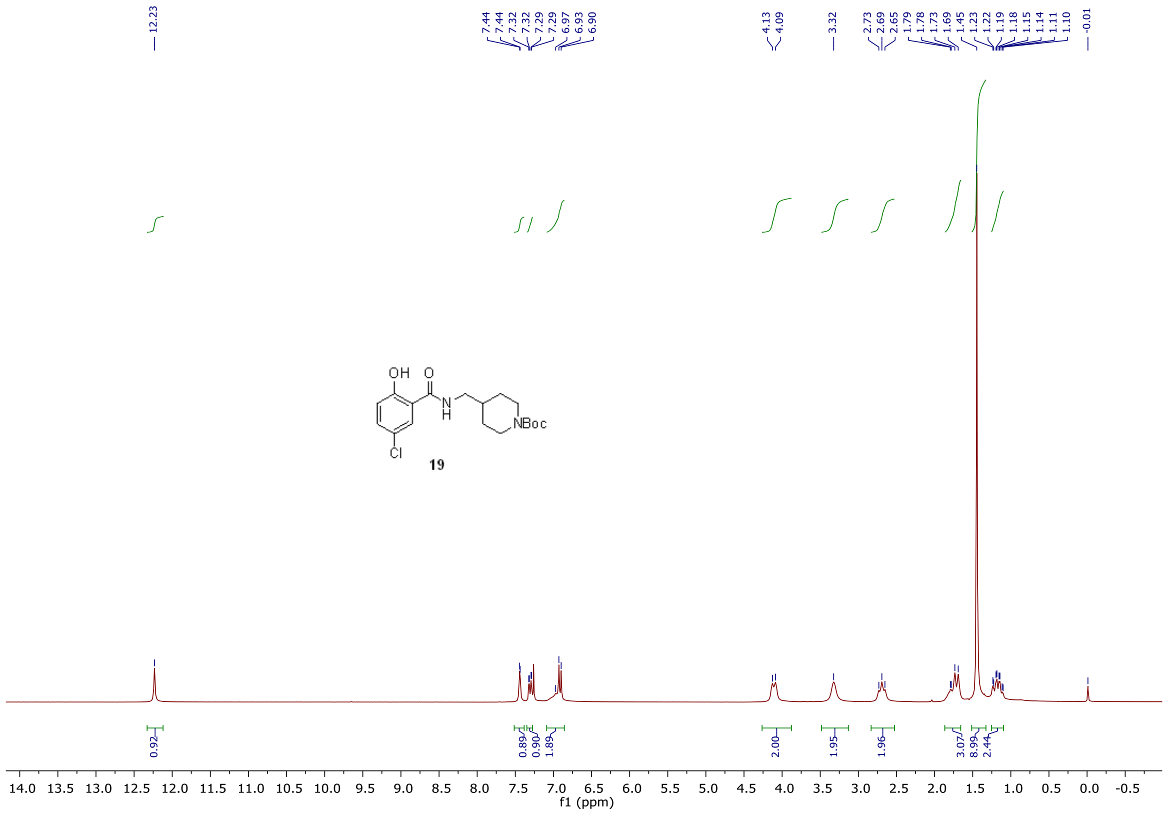
12.23
7.44 7.44 7.32 7.32 7.29 7.29 6.97 6.93 6.90
4.13 4.09
3.32
2.73 2.69 2.65 1.79 1.78 1.73 1.69 1.45 1.23 1.22 1.19 1.18 1.15 1.14 1.11 1.10
-0.01
OH O
N H
NBoc
Cl
19
0.92
0.89 0.90 1.89
2.00
1.95
1.96
3.07 8.99 2.44
14.0 13.5 13.0 12.5 12.0 11.5 11.0 10.5 10.0 9.5 9.0 8.5 8.0 7.5 7.0 6.5 6.0 5.5 5.0 4.5 4.0 3.5 3.0 2.5 2.0 1.5 1.0 0.5 0.0 -0.5
f1 (ppm)

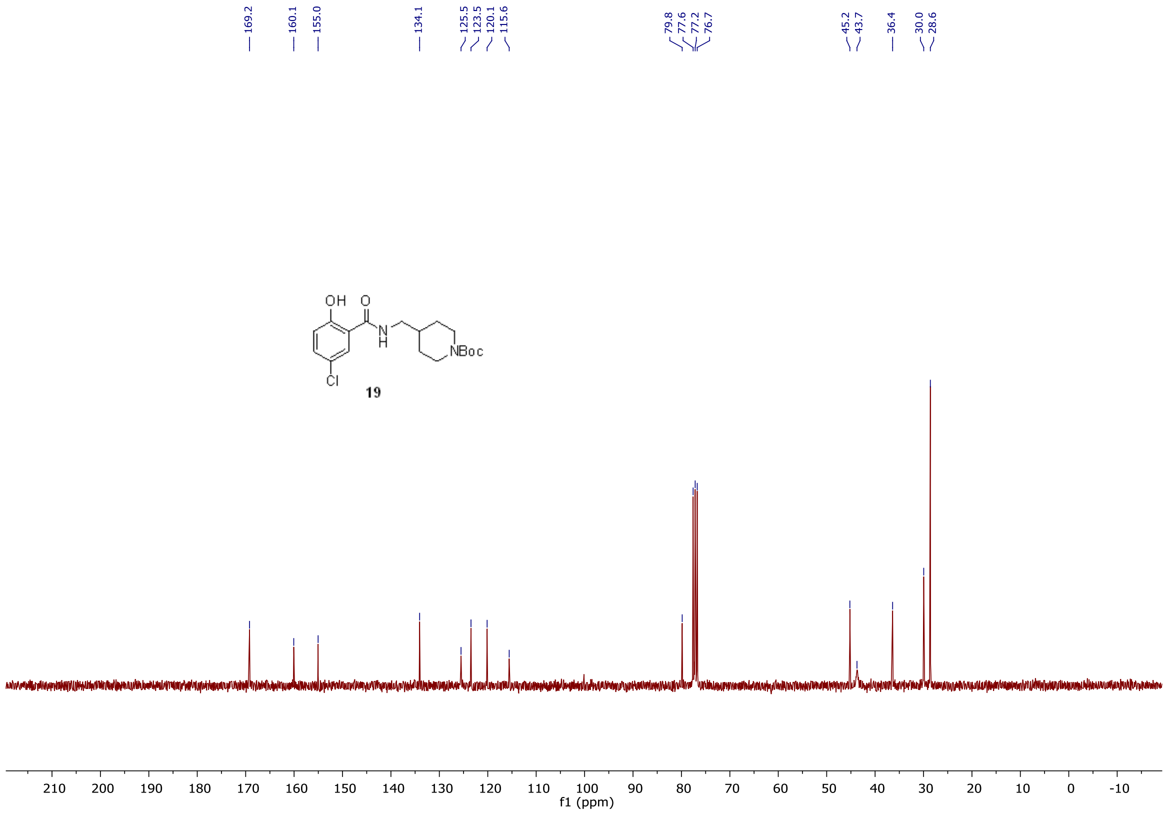
169.2
160.1
155.0
134.1
125.5 123.5 120.1 115.6
79.8 77.6 77.2 76.7
45.2 43.7
36.4
30.0 28.6
OH O
N H
NBoc
Cl
19
210 200 190 180 170 160 150 140 130 120 110 100 90 80 70 60 50 40 30 20 10 0 -10
f1 (ppm)

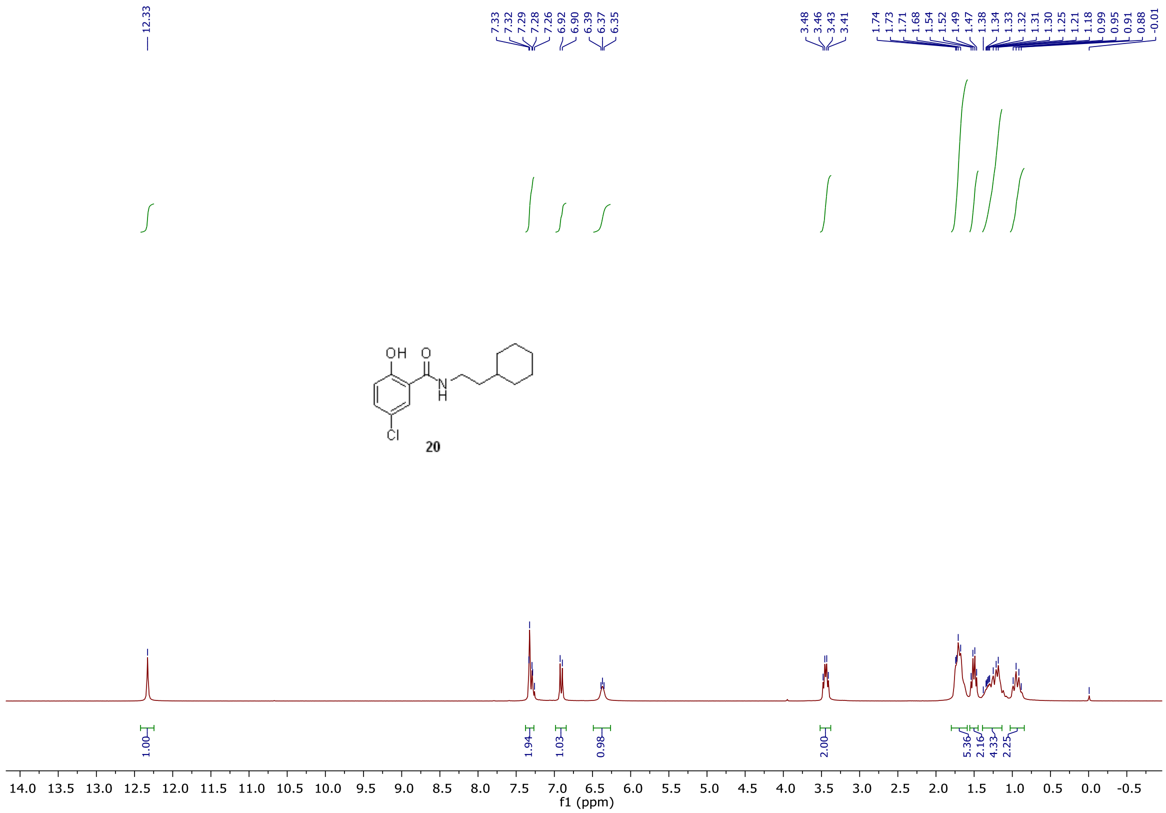

20

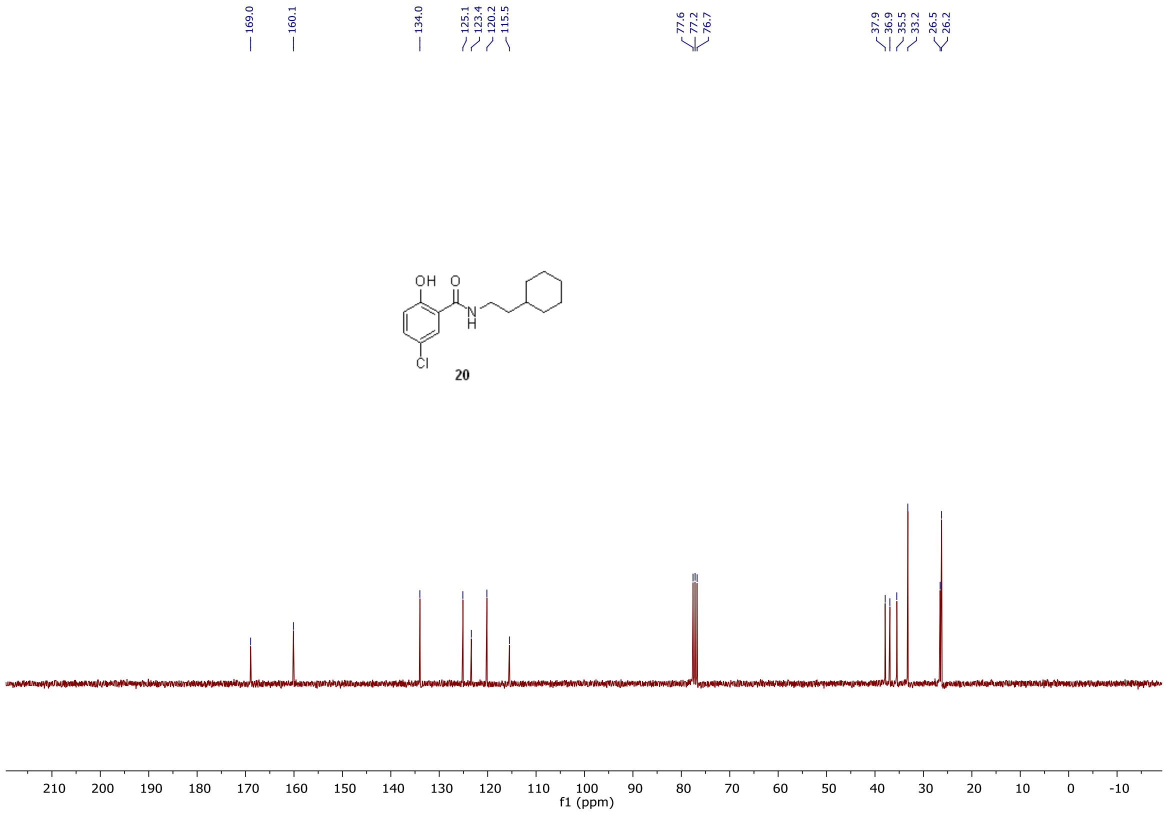

20

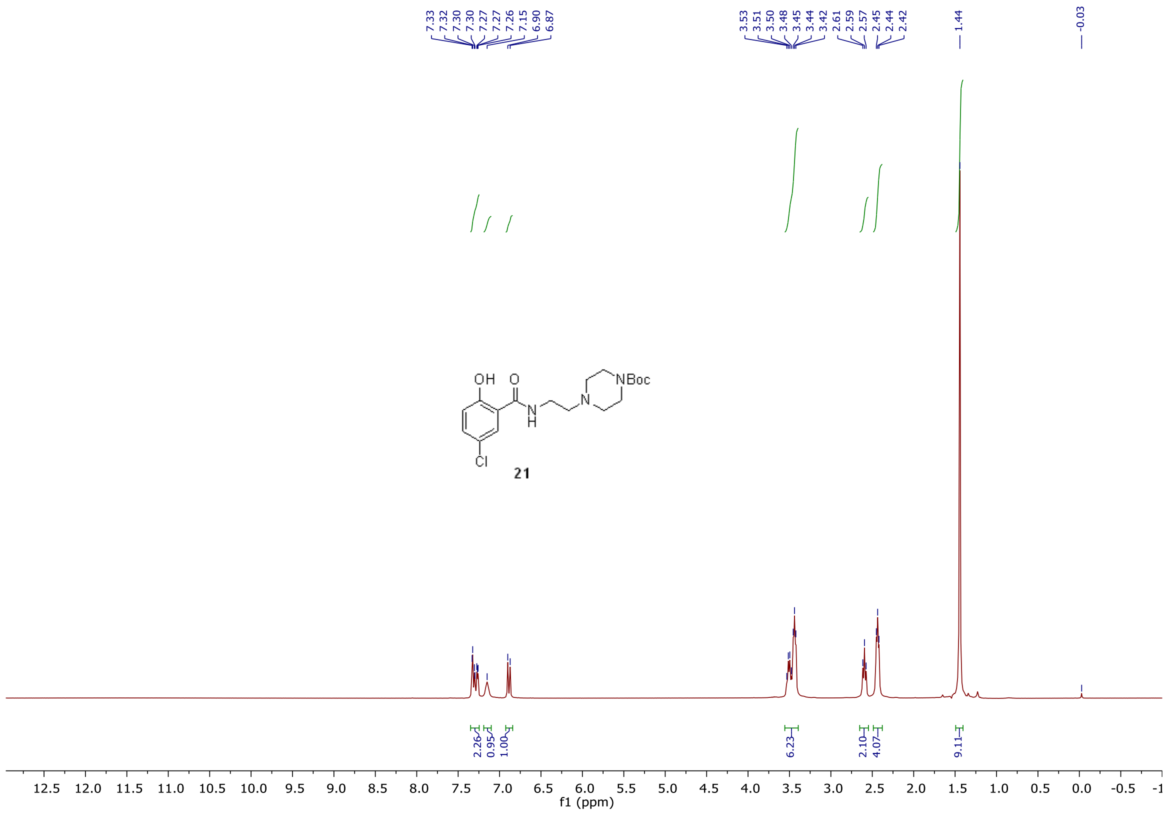
7.33
7.32
7.30
7.27
7.27
7.26
7.15
6.90
6.87
3.53
3.51
3.50
3.48
3.45
3.44
3.42
2.61
2.59
2.57
2.45
2.44
2.42
1.44
-0.03
OH O
NBoc
N
H
N
Cl
21
2.26
0.95
1.00
6.23
2.10
4.07
9.11
f1 (ppm)

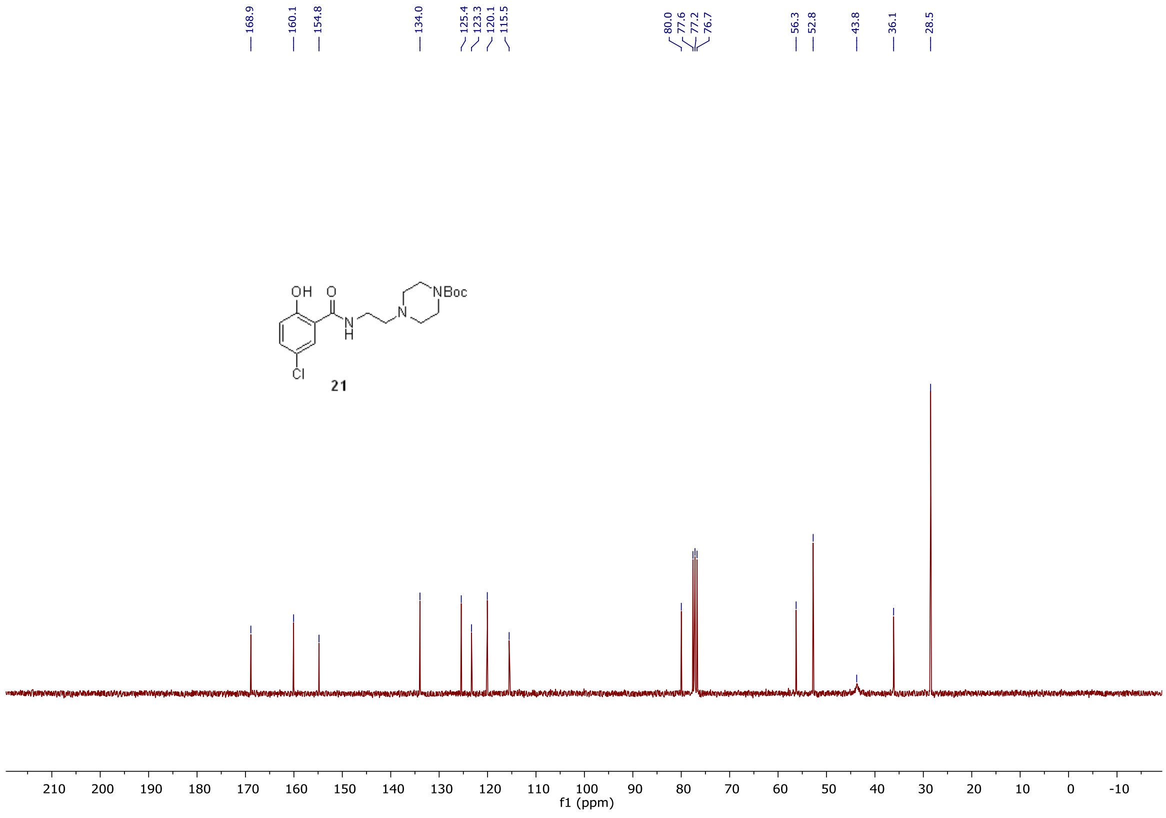
168.9
160.1
154.8
134.0
125.4
123.3
120.1
115.5
80.0
77.6
77.2
76.7
56.3
52.8
43.8
36.1
28.5
OH O
NBoc
N
H
N
Cl
21
f1 (ppm)

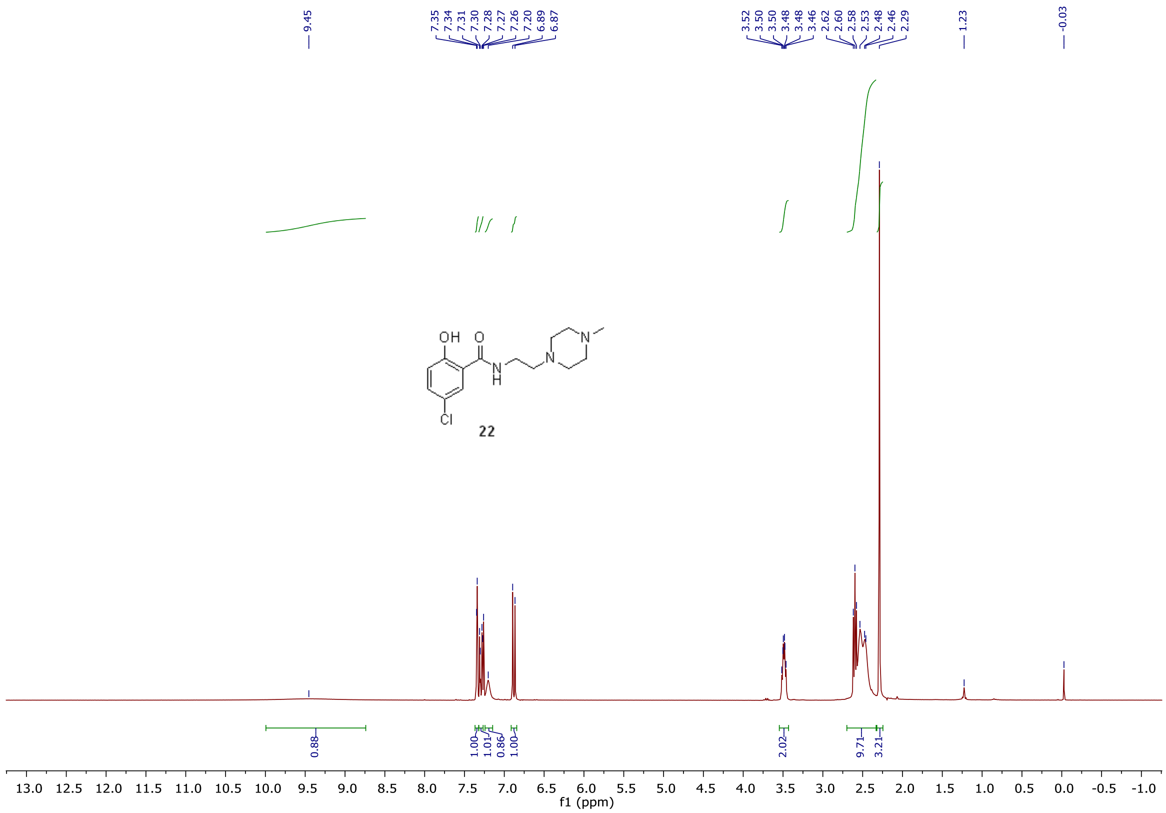
9.45
7.35 7.34 7.31 7.30 7.28 7.27 7.26 7.20 6.89 6.87
3.52 3.50 3.50 3.48 3.48 3.46 2.62 2.60 2.58 2.53 2.48 2.46 2.29
1.23
-0.03
OH O
N
H
N
N
Cl
22
0.88
1.00 1.01 0.86 1.00
2.02
9.71 3.21
13.0 12.5 12.0 11.5 11.0 10.5 10.0 9.5 9.0 8.5 8.0 7.5 7.0 6.5 6.0 5.5 5.0 4.5 4.0 3.5 3.0 2.5 2.0 1.5 1.0 0.5 0.0 -0.5 -1.0
f1 (ppm)

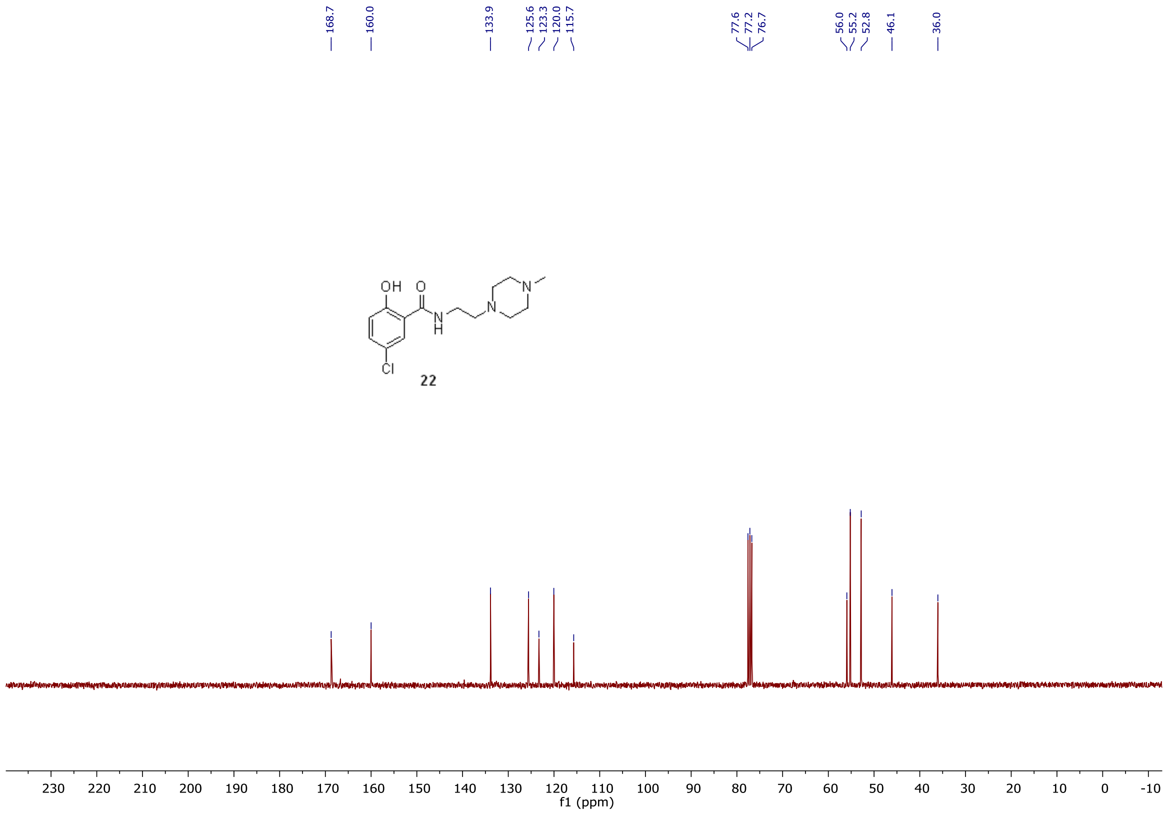
168.7
160.0
133.9
125.6 123.3 120.0 115.7
77.6 77.2 76.7
56.0 55.2 52.8
46.1
36.0
OH O
N
H
N
N
Cl
22
230 220 210 200 190 180 170 160 150 140 130 120 110 100 90 80 70 60 50 40 30 20 10 0 -10
f1 (ppm)

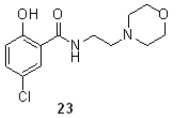

23

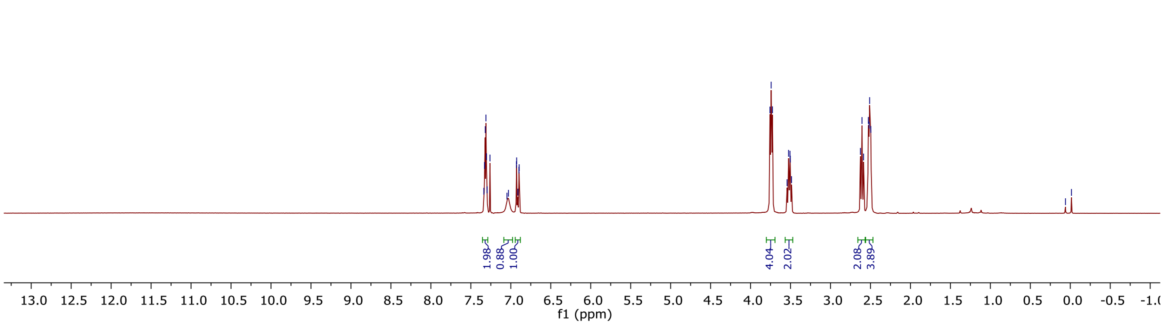

7.34 7.33 7.32 7.31 7.30 7.30 7.26 7.05 7.03 6.93 6.93 6.91 6.90 6.89
3.76 3.74 3.73 3.54 3.52 3.51 3.50 3.49 2.63 2.61 2.59 2.53 2.52 2.51 2.50
0.06 -0.01
1.98 0.88 1.00 4.04 2.02 2.08 3.89
13.0 12.5 12.0 11.5 11.0 10.5 10.0 9.5 9.0 8.5 8.0 7.5 7.0 6.5 6.0 5.5 5.0 4.5 4.0 3.5 3.0 2.5 2.0 1.5 1.0 0.5 0.0 -0.5
f1 (ppm)

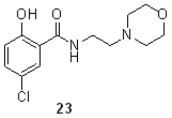

23

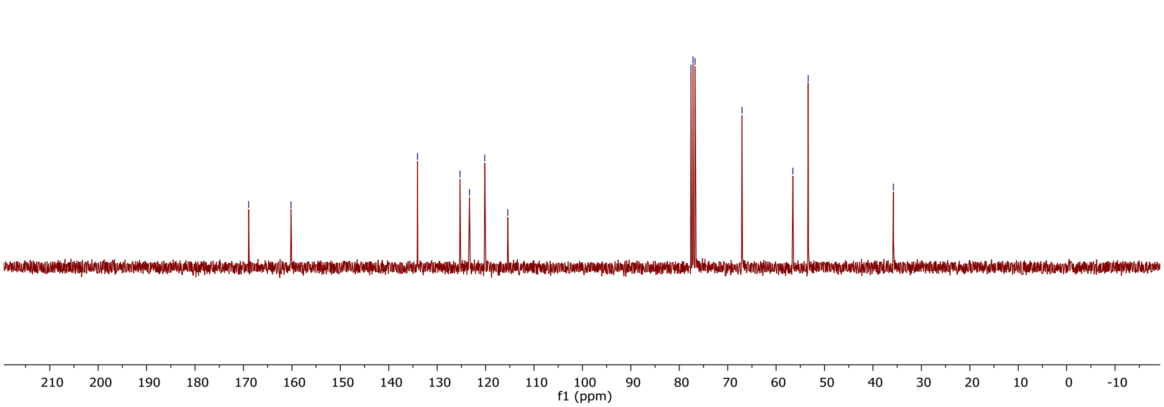

168.9 160.2 134.1 125.3 123.3 120.2 115.4 77.6 77.2 76.7 67.1 56.5 53.4 35.8
210 200 190 180 170 160 150 140 130 120 110 100 90 80 70 60 50 40 30 20 10 0 -10
f1 (ppm)

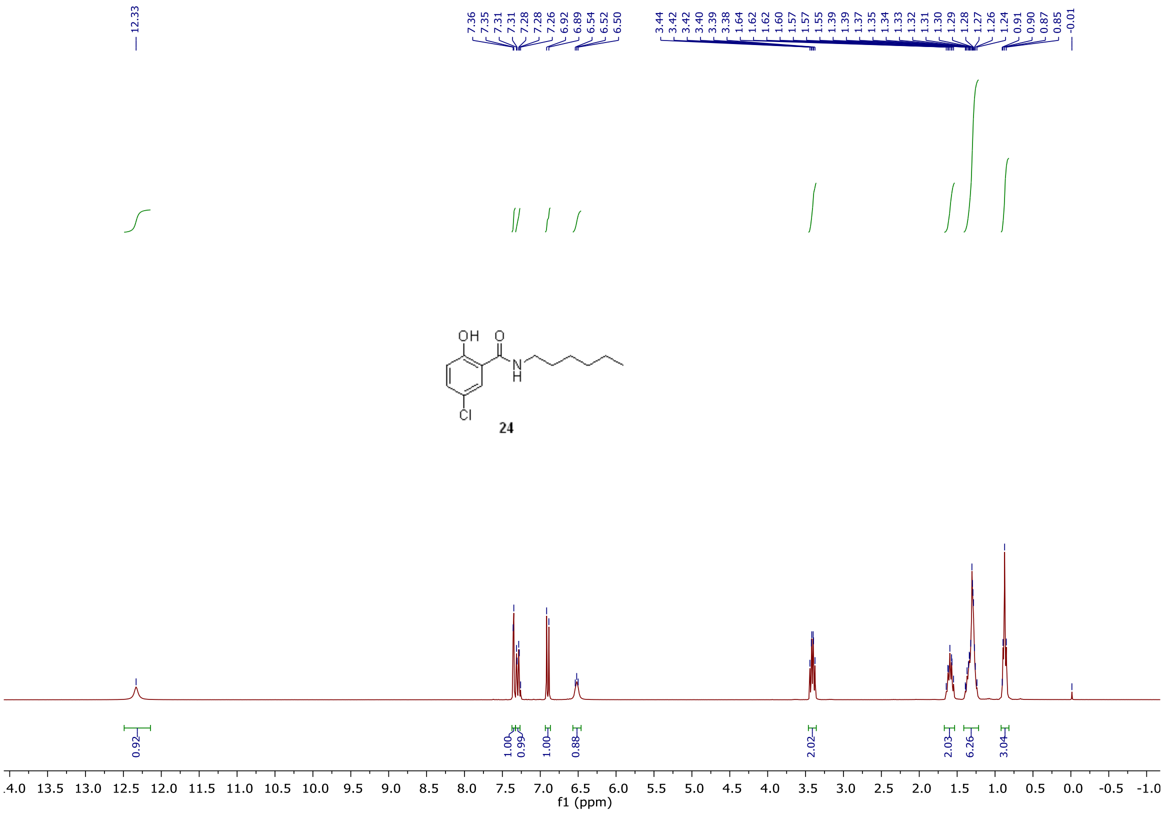

24

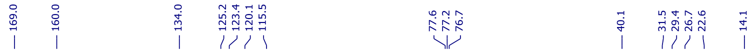

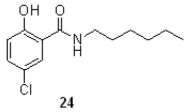

24

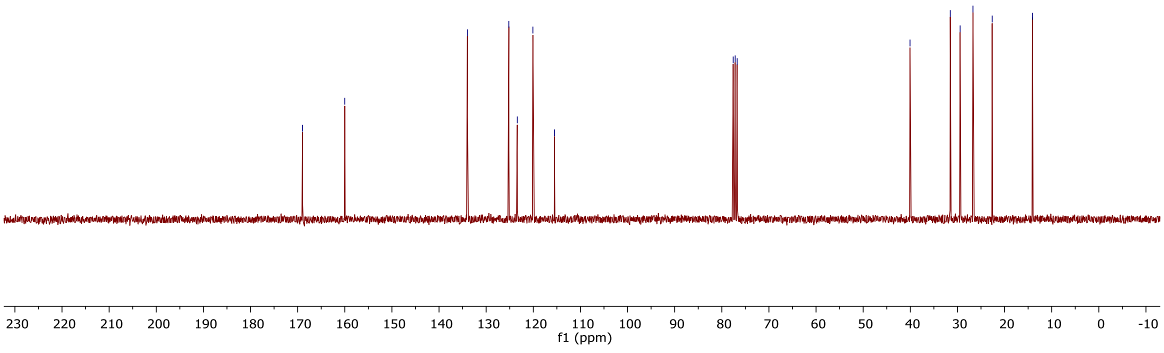

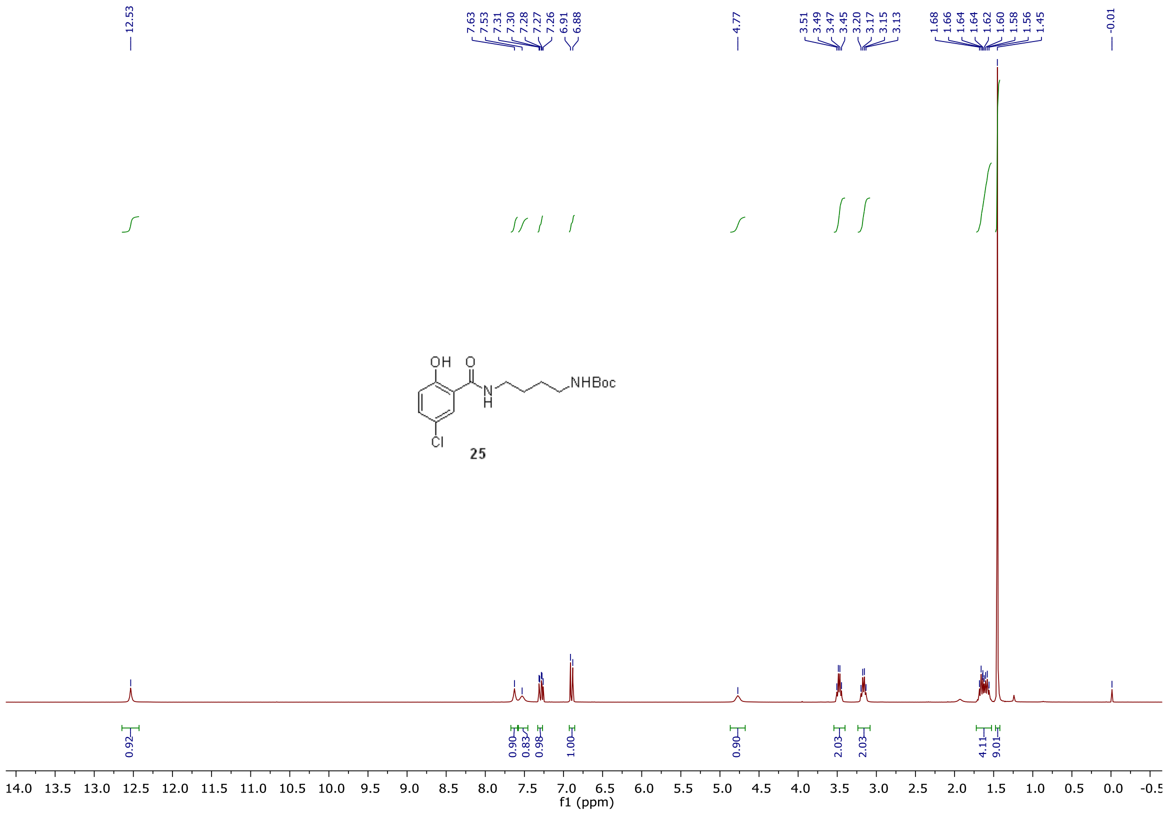
12.53
7.63 7.53 7.31 7.30 7.28 7.27 7.26 6.91 6.88
4.77
3.51 3.49 3.47 3.45 3.20 3.17 3.15 3.13
1.68 1.66 1.64 1.64 1.62 1.60 1.58 1.56 1.45
-0.01
OH
O
N
H
NHBoc
Cl
25
0.92
0.90 0.83 0.98
1.00
0.90
2.03
2.03
4.11
9.01
14.0 13.5 13.0 12.5 12.0 11.5 11.0 10.5 10.0 9.5 9.0 8.5 8.0 7.5 7.0 6.5 6.0 5.5 5.0 4.5 4.0 3.5 3.0 2.5 2.0 1.5 1.0 0.5 0.0 -0.5
f1 (ppm)

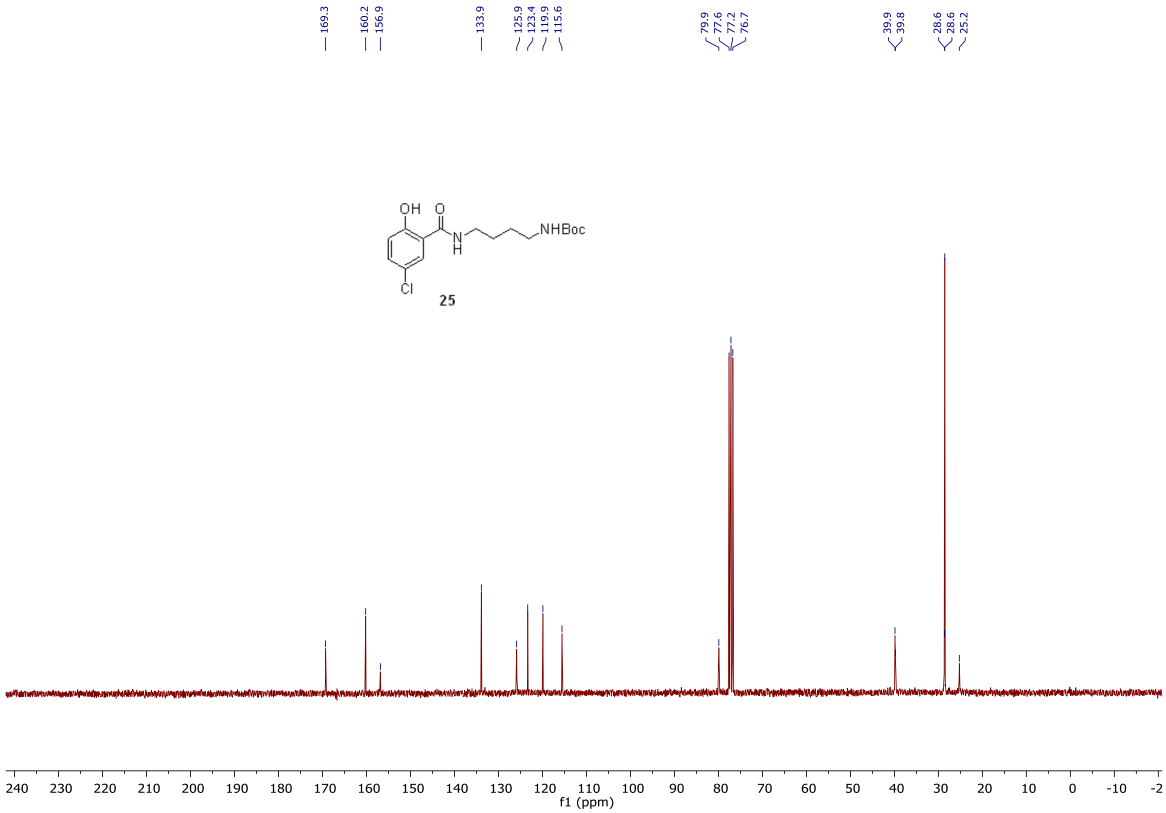
169.3
160.2 156.9
133.9
125.9 123.4 119.9 115.6
79.9 77.6 77.2 76.7
39.9 39.8
28.6 28.6 25.2
OH
O
N
H
NHBoc
Cl
25
240 230 220 210 200 190 180 170 160 150 140 130 120 110 100 90 80 70 60 50 40 30 20 10 0 -10 -2
f1 (ppm)

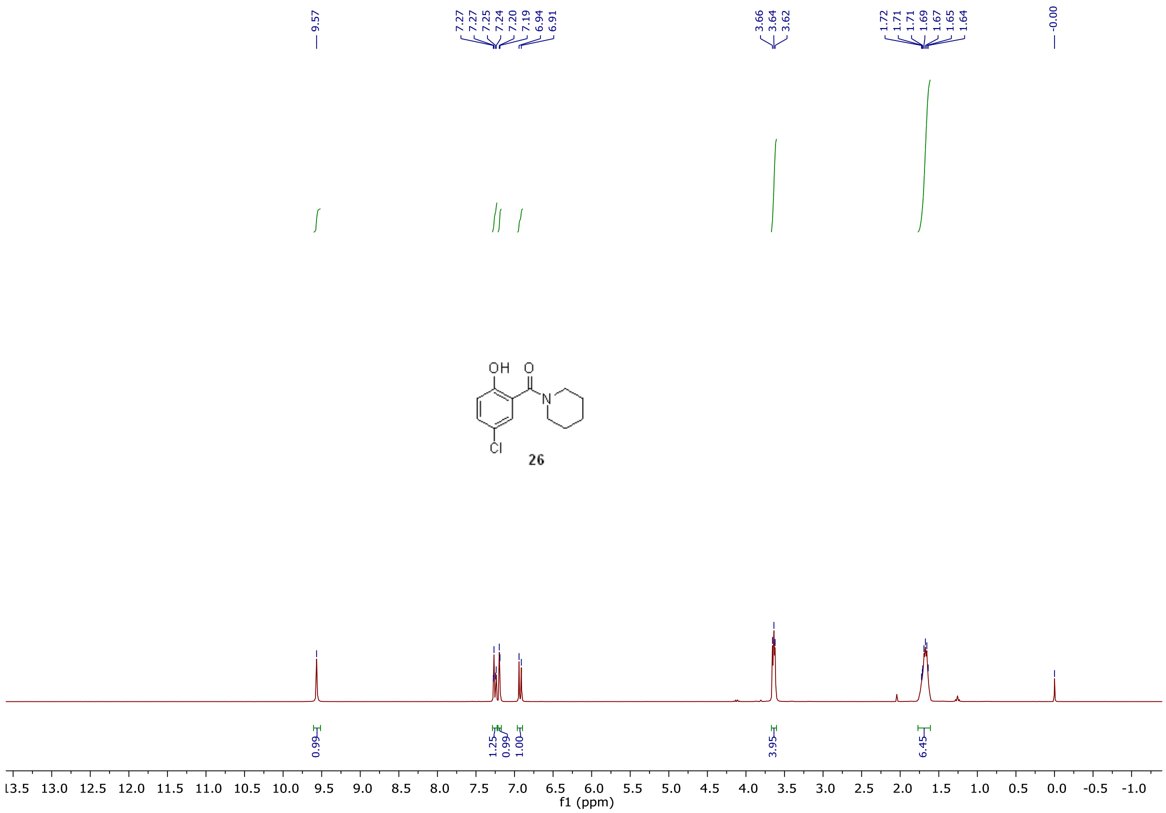
9.57
7.27
7.27
7.25
7.24
7.20
7.19
6.94
6.91
3.66
3.64
3.62
1.72
1.71
1.71
1.69
1.67
1.65
1.64
-0.00
OH
O
N
Cl
26
0.99
1.25
0.99
1.00
3.95
6.45
f1 (ppm)

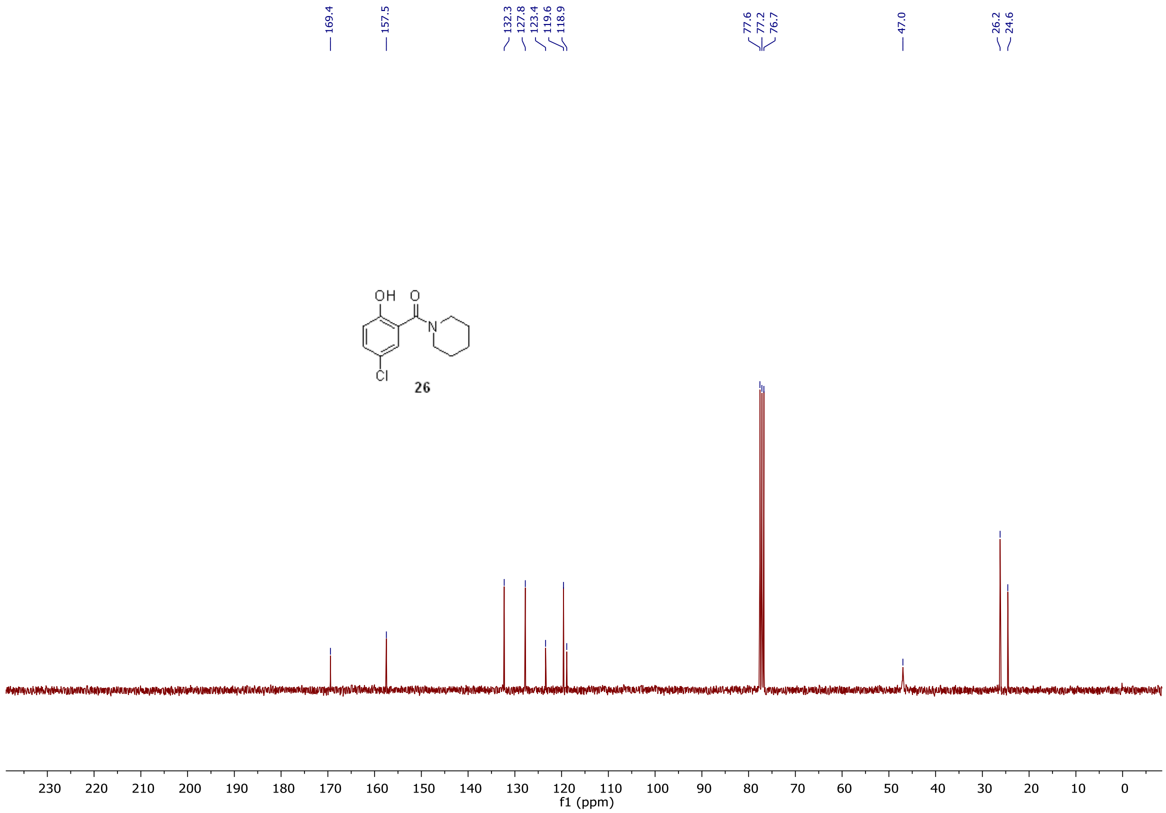
169.4
157.5
132.3
127.8
123.4
119.6
118.9
77.6
77.2
76.7
47.0
26.2
24.6
OH
O
N
Cl
26
f1 (ppm)

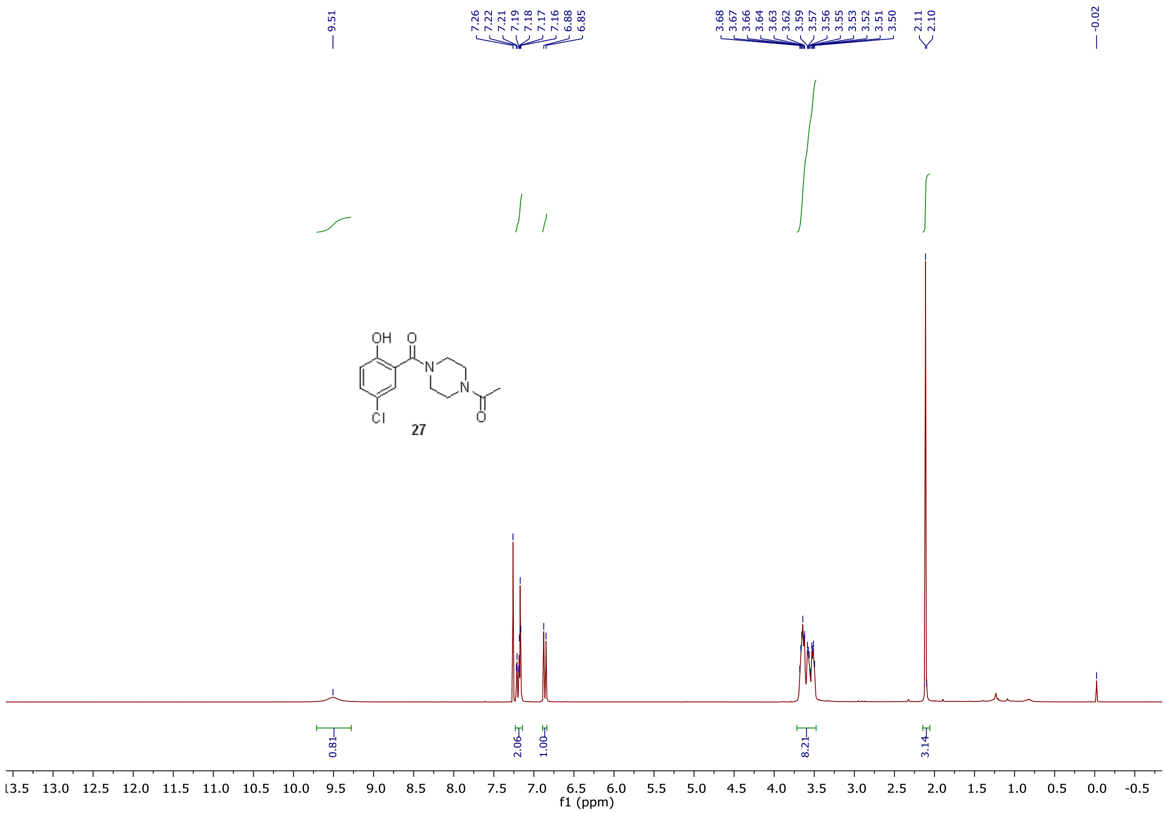
9.51
7.26 7.22 7.21 7.19 7.18 7.17 7.16 6.88 6.85
3.68 3.67 3.66 3.64 3.63 3.62 3.59 3.57 3.56 3.55 3.53 3.52 3.51 3.50
2.11 2.10
-0.02
OH
O
N
N
O
Cl
27
0.81
2.06
1.00
8.21
3.14
13.5 13.0 12.5 12.0 11.5 11.0 10.5 10.0 9.5 9.0 8.5 8.0 7.5 7.0 6.5 6.0 5.5 5.0 4.5 4.0 3.5 3.0 2.5 2.0 1.5 1.0 0.5 0.0 -0.5
f1 (ppm)

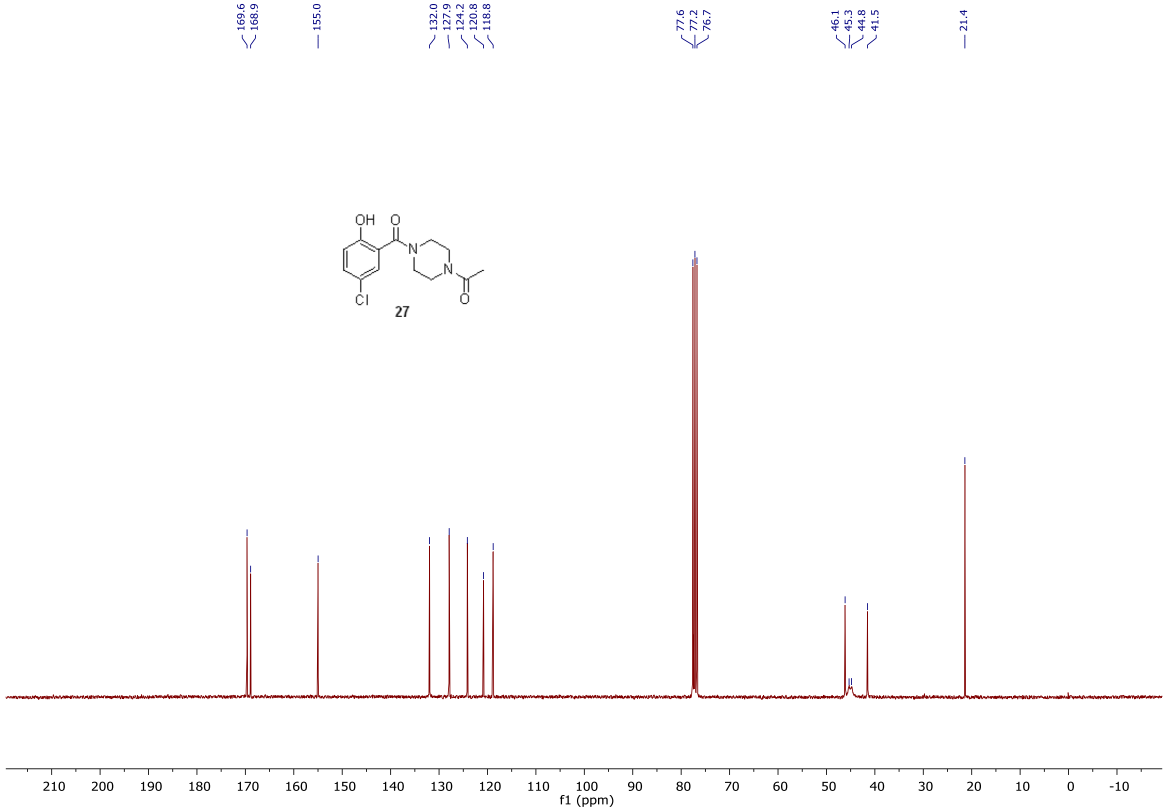
169.6 168.9
155.0
132.0 127.9 124.2 120.8 118.8
77.6 77.2 76.7
46.1 45.3 44.8 41.5
21.4
OH
O
N
N
O
Cl
27
210 200 190 180 170 160 150 140 130 120 110 100 90 80 70 60 50 40 30 20 10 0 -10
f1 (ppm)

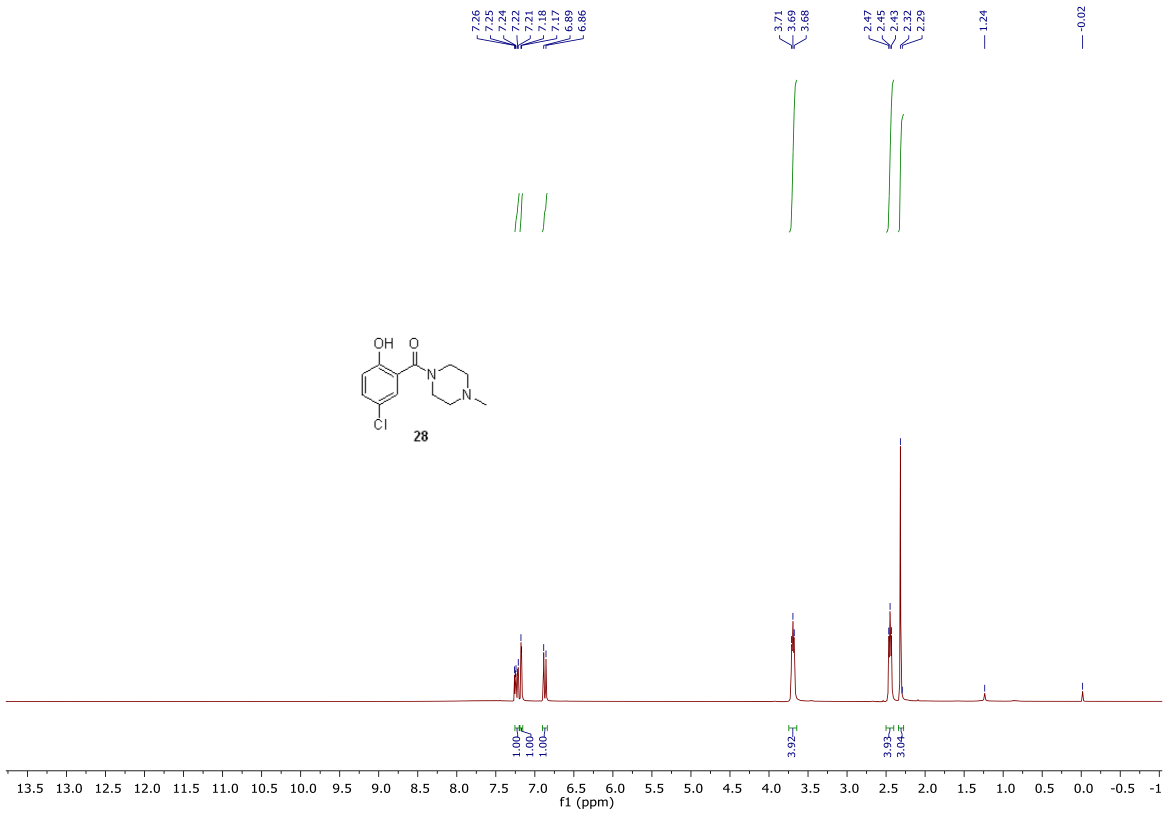
7.26 7.25 7.24 7.22 7.21 7.18 7.17 6.89 6.86
3.71 3.69 3.68
2.47 2.45 2.43 2.32 2.29
1.24
-0.02
OH
O
N
N
Cl
28
1.00
1.00
1.00
3.92
3.93
3.04
13.5 13.0 12.5 12.0 11.5 11.0 10.5 10.0 9.5 9.0 8.5 8.0 7.5 7.0 6.5 6.0 5.5 5.0 4.5 4.0 3.5 3.0 2.5 2.0 1.5 1.0 0.5 0.0 -0.5 -1
f1 (ppm)

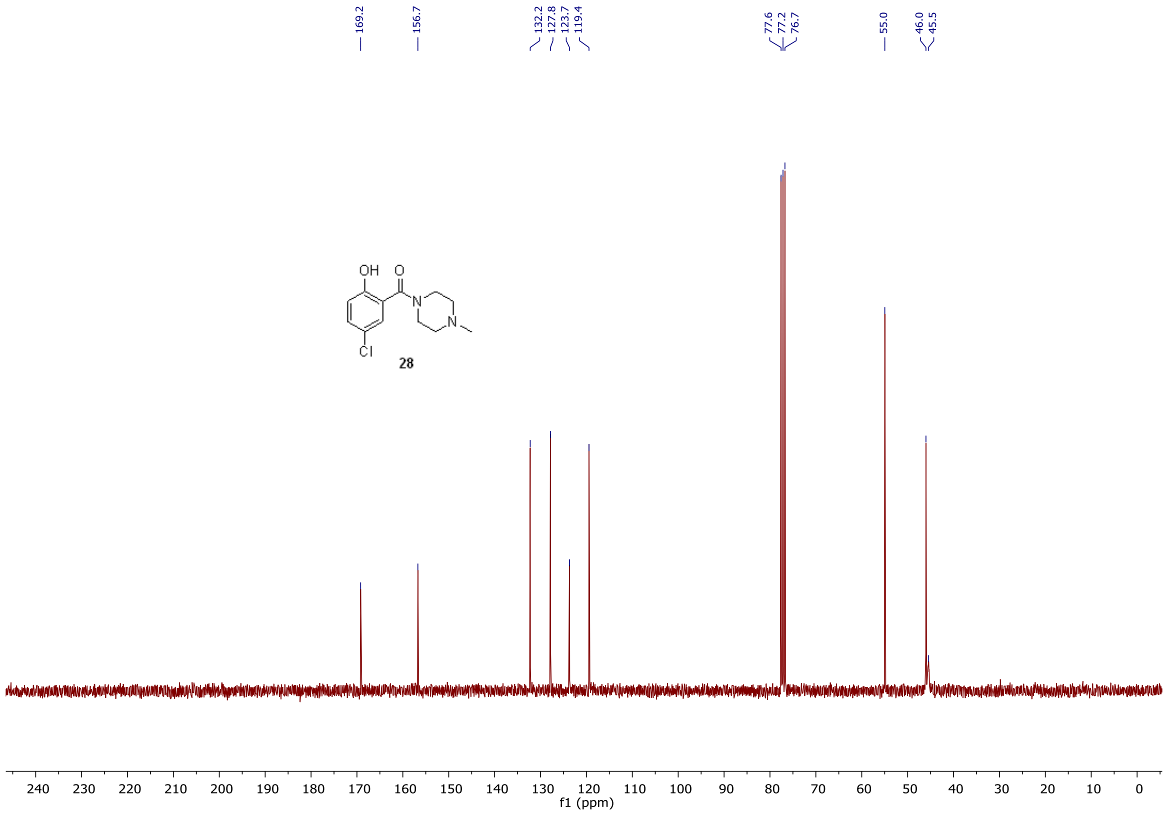
169.2
156.7
132.2 127.8 123.7 119.4
77.6 77.2 76.7
55.0
46.0 45.5
OH
O
N
N
Cl
28
240 230 220 210 200 190 180 170 160 150 140 130 120 110 100 90 80 70 60 50 40 30 20 10 0
f1 (ppm)
